# Identification of Autophagy-Associated Biomarkers and Corresponding Regulatory Factors in the Progression of Colorectal Cancer

**DOI:** 10.3389/fgene.2020.00245

**Published:** 2020-03-18

**Authors:** Chunrui Zhang, Jing Jiang, Liqiang Wang, Liyu Zheng, Jiankai Xu, Xiaolin Qi, Huiying Huang, Jianping Lu, Kongning Li, Hong Wang

**Affiliations:** ^1^College of Bioinformatics Science and Technology, Harbin Medical University, Harbin, China; ^2^Institute of Genetics and Developmental Biology, Chinese Academy of Sciences, Beijing, China; ^3^Obstetrics and Gynecology Department, The Second Affiliated Hospital of Harbin Medical University, Harbin, China; ^4^Key Laboratory of Tropical Translational Medicine of Ministry of Education and College of Biomedical Information and Engineering, Hainan Medical University, Haikou, China

**Keywords:** autophagy, colorectal cancer, regulatory network, RNA-binding proteins, biomarkers

## Abstract

Autophagy is a self-degradation process that maintains homeostasis against stress in cells. Autophagy dysfunction plays a central role in the development of tumors, such as colorectal cancer (CRC). In this study, autophagy-related differentially expressed genes, their downstream functions, and upstream regulatory factors including RNA-binding proteins (RBP) involved in programmed cell death in the CRC were investigated. Transcription factors (TFs) and miRNAs have been shown to mainly regulate autophagy genes. Interestingly, we found that some of the RBP in the CRC, such as DDX17, SETDB1, and POLR3A, play an important regulatory role in maintaining autophagy at a basal level during growth by acting as TFs that regulate autophagy. Promoter methylations showed negative regulations on differentially expressed autophagy gene (DEAG), while copy number variations revealed a positive role in them. A proportional hazards regression analysis indicated that using autophagy-related prognostic signature can divide patients into high-risk and low-risk groups. Autophagy associated FDA-approved drugs were studied by a prognostic network. This would contribute to the identifications of new potential molecular therapeutic targets for CRC.

## Introduction

Colorectal cancer (CRC) is a common digestive tract tumor (Chisanga et al., 2016). Among all cancer types, it is the third leading cause of death in the world. The overall 5-year survival rate of CRC patients is less than 40%, and the occurrence of CRC is consistently rising (Yang et al., 2015). However, the prognosis and therapy for CRC have not been significantly improved. Therefore, a proper selection of patients for aggressive treatment is necessary, new therapeutic strategies and prediction of prognosis of CRC is urgently needed.

Autophagy has been found to be associated with a variety of clinically relevant diseases, such as CRC. In the past ten years, autophagy has received extensive attention as a new treatment method. Several studies indicate that the autophagy function plays a critical role in the development, maintenance, and progression of CRC (Yang et al., 2015; Katheder et al., 2017). The dysregulation of autophagy function disrupts the physiological processes and has been implicated in the pathogenesis of multiple diseases (Thorburn et al., 2014). Early efforts reported that there are relationships between multidimensional factors and autophagy function. BECN1 plays a key role in the autophagic process, its expression is found to be regulated by transcription factors (TFs), miRNAs, the abnormal methylation of the promoter region, and copy number variation (CNV) of the associated chromatin regions (Mei et al., 2016). In addition, RNA-binding proteins (RBP) play a key role in many processes as TF, including cellular differentiation, autophagy, apoptosis, and DNA repair (Gerstberger et al., 2014; Williams et al., 2019). For instance, some researches have shown that CELF2 RNA-binding protein regulates autophagy-mediated CRC cell death (New et al., 2019). Furthermore, Kudinov AE et al. found that MSI2 RNA-binding protein as a regulator of progenitor cell is elevated in colorectal adenocarcinomas and that its loss of function inhibits the growth of CRC cells (New et al., 2019). In the past decade, autophagy as a new therapeutics has attracted extensive attention. Increasing evidence indicates that autophagy function is crucial to tumor cell survival in CRC patients undergoing anticancer treatment (Roy and Debnath, 2010). Despite this, the potential values of some novel prognostic biomarkers related to autophagy function have not been thoroughly investigated. This study will focus on the potential prognostic roles of autophagy-related genes in CRC and will offer new targets for the treatment of CRC. Further understanding of the functional role of autophagy in CRC pathogenesis will allow us to improve the disease management.

In this study, the function of autophagy genes in four stages of CRC was investigated through the performance of functional enrichment analysis of the downstream RNAs. The upstream regulatory factors of autophagy genes were also identified in each stage by integrating multi-omics data in TCGA. Some key autophagy-related differentially expressed genes associated with the prognosis of CRC were identified through univariate Cox proportional hazards regression model. Then the mappings were drawn between FDA-approved drugs and their related autophagy gene. These findings not only shed light on the central functional role of autophagy-related genes in CRC, but may also contribute to the identification of molecular biomarkers in CRC and the development of clinical therapeutic modality.

## Materials and Methods

### Colorectal Cancer Patient Cohorts

Gene and miRNA expression data, methylation data, and the clinical data of CRC patients were downloaded from TCGA^1^ (Cancer Genome Atlas Research Network, 2008). There are 328 colon carcinoma (COAD) samples and 105 rectal carcinomas (READ) samples. Combined with clinical information, there are 41 normal patients, 45 stage I patients, 111 stage II patients, 83 stage III patients, and 39 stage IV patients in COAD samples. And there are 10 normal patients, 12 stage I patients, 28 stage II patients, 33 stage III patients, and 15 stage IV patients in READ samples. The corresponding CNV data were obtained from the Cancer Cell Line Encyclopedia^2^ (Barretina et al., 2012). Additionally, a cohort of 177 COAD patients and 196 READ patients from the GEO database (GSE17536 and GSE87211) (Smith et al., 2010; Hu et al., 2018) was used as an independent external test set.

### Autophagy Genes, Interaction Data, RNA-Binding Proteins, and Transcription Start Sites

Autophagy genes were collected from the cell death proteomics database^3^ (Arntzen et al., 2013). A total of 1776 experimentally confirmed genes were used for the subsequent analysis. Protein-protein interactions were retrieved from the Human Protein Reference Database (HPPD)^4^ (Keshava Prasad et al., 2009). The TF that targeted the autophagy genes were acquired from ChIPBase^5^ (Zhou et al., 2017). The 2949 RBP were downloaded from the EuRBPDB^6^ (New et al., 2019). The miRNA-gene targeted interaction was formed through the integration of miRecords^7^ (Xiao et al., 2009), DIANA-TarBase^8^ (Vlachos et al., 2015), and miRTarBase^9^ (Chou et al., 2018) databases.

The transcription start sites (TSS) of autophagy genes were downloaded from GENCODE (Harrow et al., 2012). The mean of the methylation level for CG sites in autophagy gene transcription promoter regions was used as the methylation level of the autophagy genes.

### Construction of Regulatory Networks and the Influence of Regulators on the Differentially Expressed Autophagy Gene

The significant differentially expressed autophagy gene (DEAG) and regulated gene pairs were obtained through the calculation of their linear correlation based on the expression data (*P* < 0.05). Linear regression was then used to calculate the significant TFs and miRNAs that targeted the DEAG based on known TF/miRNA-gene interaction (*P* < 0.05). The significant influence of CNVs or gene promoter methylations was denoted by the linear correlation between gene expression and their own CNVs or gene promoter methylations level (*P* < 0.05).

### Statistical Analysis

The relationship between DEAGs expression level and patient survival was evaluated by the Cox regression analysis. Multivariate Cox regression analysis was used to fit the selected DEAGs (Lossos et al., 2004). The risk score of each patient was calculated with the estimated regression coefficient as the weight (Zhou et al., 2016). It was calculated as follows:

$$\text{Risk}\,\_\,\text{score}=\sum_{i=1}^{n}\beta_{i}\times {\text{EXP}}_{\text{gene}\,\left(i\right)}$$

where β_*i*_ is the Cox regression coefficient of gene *i* in the training set, and *n* is the number of survival related genes. The sensitivity and specificity of survival gene risk prediction were compared using the time-dependent receiver operating characteristic (ROC) curves, and the optimal patient stratified cutoff value was determined in the discovery cohort. Patients were divided into high risk group and low risk group in accordance with the above stratification cutoff. Kaplan–Meier survival analysis and log-rank test were performed to compare survival differences. Cox proportional risk regression was used for multivariate analysis to test whether the autophagy gene signature was independent of other clinic-pathological factors. The Cox proportional risk regression model was used to estimate the hazard ratio (HR) and the 95% confidence intervals (CI).

## Results

### Construction of DEAG Regulatory Network

**FIGURE 1:**
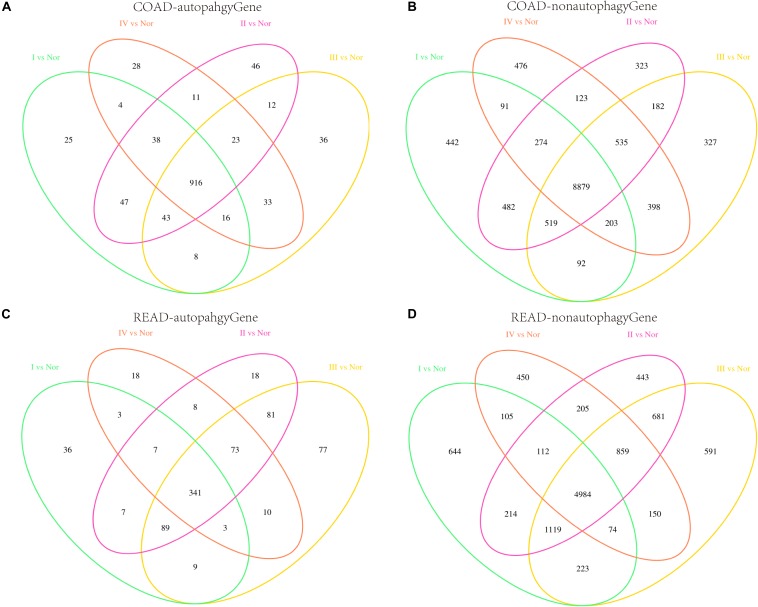
Differentially expressed genes in the four stages. **(A,B)** Represent the Venn diagrams of differentially expressed autophagy genes and non-autophagy genes respectively in four stages of COAD. **(C,D)** Represent the Venn diagrams of differentially expressed autophagy genes and non-autophagy genes respectively in four stages of READ.

Genome-wide analysis of mRNA expression was performed to identify differentially expressed mRNAs, and the autophagy genes were extracted. There were 1097, 1136, 1087, and 1069 DEAGs between the normal and each stage (I, II, III, and IV) cancer samples in COAD. There were 495, 624, 683, and 463 DEAGs in READ (*P* < 0.05). It was found that a large proportion of the DEAGs were shared among the four stages (Figures 1A,C). Used Chi-square Test, we found the four stages significantly shared the majority of the DEAGs. The *P*-values of each stage of COAD are 0.03601, 0.09514, 0.00041, and 0.00014, respectively. Genes which appeared in three, two and single stages were infrequent. By contrast differentially expressed non-autophagy genes that only appeared in a single stage were most common (Figures 1B,D). We identified 12079, 12453, 12222, and 12048 differentially expressed genes between the normal and each stage (I, II, III, and IV) cancer samples in COAD. And there were 7970, 9241, 9364, and 7402 differentially expressed genes in READ. The autophagy genes were extracted, there were 1097, 1136, 1087, and 1069 DEAGs in COAD and 495, 624, 683, and 463 DEAGs in READ. So differentially expressed non-autophagy genes were 10982, 11317, 11135, and 10979 in COAD and 7475, 8617, 8681, and 6939 in READ. These results imply that autophagy genes play an important role during the development and progression of CRC. Whereas a few autophagy genes, which were expressed differently in specific stages of cancer, reflect that these genes play a different role in different stages.

**FIGURE 2:**
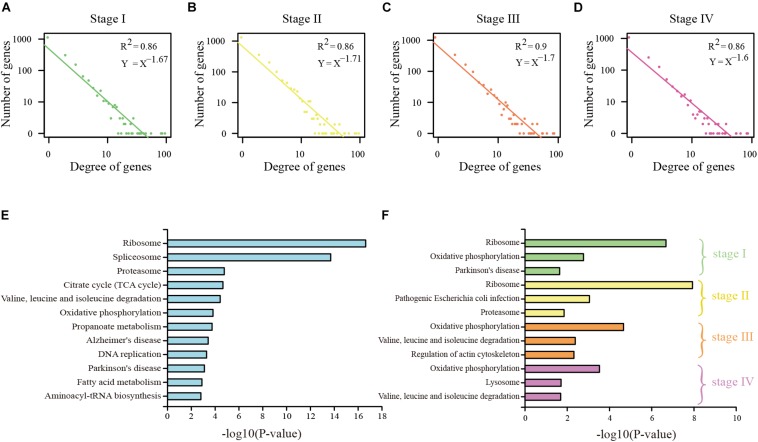
Analysis of DEAG regulatory network in COAD. **(A)** The distribution of the degree of DEAG regulatory network in stage I. **(B)** The distribution of the degree of DEAG regulatory network in stage II. **(C)** The distribution of the degree of DEAG regulatory network in stage III. **(D)** The distribution of the degree of DEAG regulatory network in stage IV. **(E)** Significantly enriched KEGG pathways of common DEAGs. **(F)** Significantly enriched KEGG pathways of the DEAGs in every stage.

To study the regulation ability of the DEAGs, the regulatory network was constructed by the linear regression method (Supplementary Figures S1, S2). Through network topology analysis, it was discovered that the networks exhibit power law degree distribution. This illustrates the scale-free and small-world nature of these networks, which makes them similar to the general biological network (Figures 2A–D and Supplementary Figures S3A–D). In conclusion, many pieces of evidence indicate that specific DEAGs and their regulatory subnetwork in each of the cancer stages can better represent the function of autophagy genes in its own stage.

Functional enrichment analysis for KEGG pathway was performed on the common DEAGs and the specific DEAGs through the use of DAVID 6.8 bioinformatics tool (Huang et al., 2009). Common DEAGs in COAD were enriched in 22 KEGG pathways (*P* < 0.05), including ribosome, spliceosome, proteasome, propanoate metabolism, and fatty acid metabolism (Figure 2E). In READ, there were spliceosome, methane metabolism, fatty acid metabolism, and propanoate metabolism (Supplementary Figure S3E). This is consistent with the findings of Y. Boglev et al. Genetic mutations associated with ribosomal production provide a powerful stimulus to autophagy in affected tissues, allowing them to escape cell death. Autophagy is a specific response to damage in ribosome organisms (Boglev et al., 2013). However, the influence of DEAGs in each distinct stage was found to be a little different (Figure 2F and Supplementary Figure S3F). Stage I and stage II were analogous, and stage III and stage IV were analogous. This reflects that the genes in stage I and stage II play a similar role, and the genes in stage III and stage IV play a similar role.

### Analysis of DEAG Regulatory Mechanism With Multi-Omics Data

**FIGURE 3:**
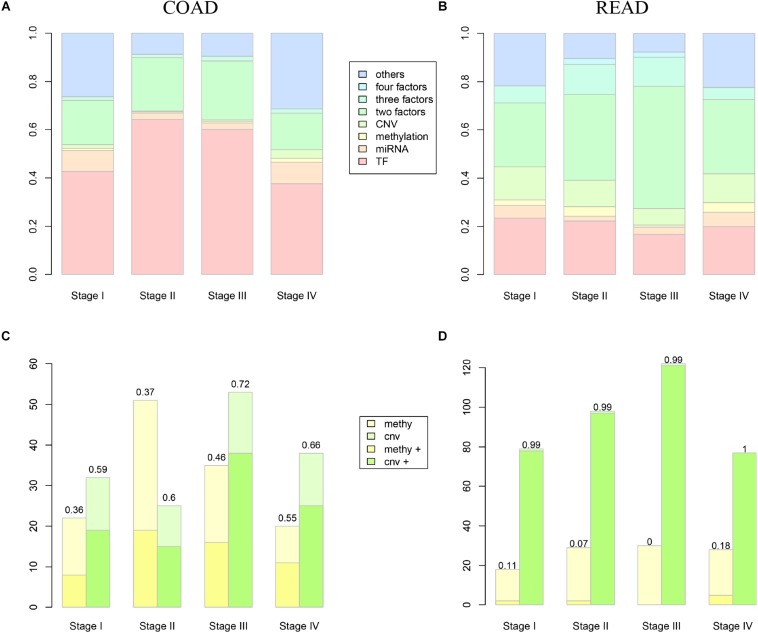
The influence of different level factors. **(A,B)** Show the proportion of DEAGs regulated by CNVs, methylation, miRNAs, TFs, and composite factors in COAD and READ respectively. **(C,D)** Show the influence of methylation and CNV to DEAGs in COAD and READ respectively. Yellow boxes represent the number of autophagy genes influenced by methylation, while green boxes represent the number of autophagy genes influenced by CNVs. The lighter color represents negative regulation, and the deeper color represents positive regulation.

**FIGURE 4:**
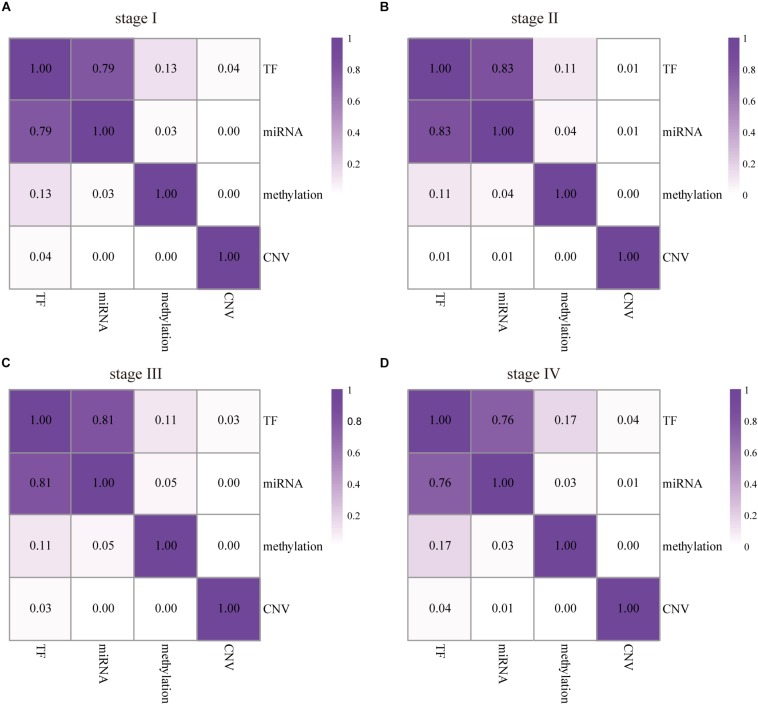
The influence of any two factors to DEAGs in the four stages of COAD. The numbers represent the proportion of the DEAGs regulated by each pair of upstream regulators in stage I **(A)**, II **(B)**, III **(C)**, and IV **(D)**. The darker color represents a larger effect.

Along with the development and maturation of the new generation sequencing technology, more and more multi-omics data could be obtained. This study primarily analyzed the impacts of CNVs, gene promoter methylations, miRNAs, and TFs on the expression of DEAG. To investigate the extent of influence, the percentage of DEAG regulated by each factor and the combination of multiple factors was calculated (Figures 3A,B, the detailed percentage of different factors for each stage was added to Supplementary Table S3). The majority of DEAGs are regulated by TFs. This is possibly due to a large amount of TFs present in the cells. The next major factor is miRNA, which negatively influenced these genes. A small number of DEAGs were affected by their own promoter CNVs and gene promoter methylations. Furthermore, a certain proportion of the DEAGs was subjected to a comprehensive regulation of multiple factors. Around 20% are jointly regulated by two factors. So, the DEAGs regulated by any two factors were thoroughly investigated (Figure 4 and Supplementary Figure S4). There are no doubts that TF and miRNA synergistically influenced a large portion of the DEAGs, which may be a result of their relatively large quantity. The influence of CNVs and gene promoter methylations cannot be ignored. Hypermethylation of gene promoter generally has a negative influence on genetic expression, and the CNVs generally has a positive influence (Figures 3C,D). This pattern is consistent with the pre-transcriptional regulation of the gene.

### Prognostic Value of the Biomarker for Survival Prediction

**FIGURE 5:**
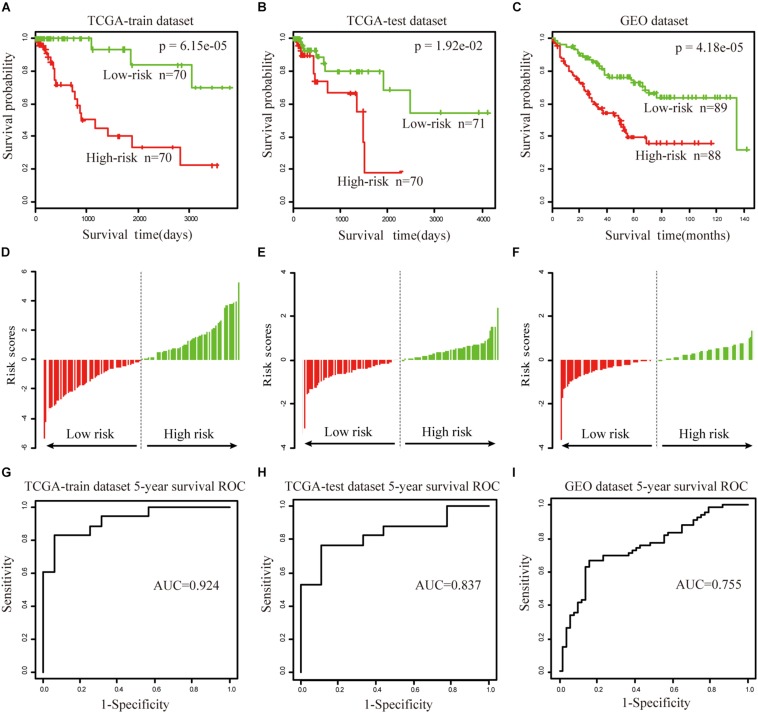
The prognostic value of the nine genes signature in COAD. **(A–C)** Show the Kaplan–Meier survival curves for the train **(A)**, test **(B)**, and GEO datasets **(C)**. The red and green lines represent the high-risk and low-risk patients respectively. *P*-value means log rank *P*. **(D–F)** Show the detailed risk score distribution of patients in the train **(D)**, test **(E)**, and GEO datasets **(F)**. **(G–I)** Show the ROC curves and AUCs of the nine gene signature predicting patients’ five-year survival in the train **(G)**, test **(H)**, and GEO datasets **(I)**.

To further validate the prognostic performance, a univariate Cox proportional hazards regression model was used to evaluate the association between the DEAGs expression levels and overall survival (OS). The 281 COAD patients were divided randomly into a train dataset (*n* = 140) and an internal test dataset (141). It was found that nine genes were significantly associated with OS in the train dataset (*P* < 0.01). Using the regression coefficients estimated in the multivariate Cox regression analysis as weights, the risk score for each patient in the train dataset was calculated by a linear combination of the expression levels of the nine-gene. These scores were classified into high-risk group (*n* = 70) and low-risk group (*n* = 70) with the median risk score as the cutoff point. The result showed that patients in the high-risk group exhibited poor OS compared with those in the low-risk group (log rank *P* < 0.05) (Figures 5A,D). A time-dependent ROC curves analysis performed on the nine-gene and the area under curve (AUC) was achieved at 0.924 (Figure 5G). These genes can effectively stratify patients into different risk groups, which suggests that they may play essential roles in COAD. Internal test datasets were used to evaluate the prognostic value of the nine-gene signatures in predicting survival (Figures 5B,E,H) and a GEO dataset (Figures 5C,F,I). Patients of the internal test dataset and GEO dataset were divided into high-risk group and low-risk group with accordance to the same nine-gene signature score model derived from the train dataset. As in the train dataset, OS of high-risk group was significantly worse than that of the low-risk group (log rank *P* < 0.01). These results demonstrated that the nine genes were potential prognostic biomarkers for the prediction of tumor risk in COAD.

The univariate and multivariate analysis indicated that the nine-gene module biomarker was significantly associated with the OS of the COAD patients in the train and internal test dataset (Table 1). Additionally, the multivariate analysis also demonstrated that the designation of high-risk and low-risk groups remained statistically significant in the independent GEO dataset. In conclusion, these analyses demonstrated the capacity of the nine-gene biomarkers for COAD, and its ability to add value in the prognostic setting. This process was then systematically executed on the study of READ (Supplementary Figure S5), there are fifteen-gene biomarkers for READ.

**TABLE 1:** Univariate and multivariate Cox regression analysis in the COAD.

Variables	Univariate analysis			Multivariate analysis		
	HR	95% CI	*P*-value	HR	95% CI	*P*-value
**Train dataset**						
Nine genes	2.795	1.894–4.125	2.258e-07	2.394	1.617–3.546	1.32e-05
Stage						
I,II	1(reference)			1(reference)		
III,IV	2.307	0.880–6.052	0.0892	2.639	0.742–9.390	0.134
Age	1.009	0.974–1.045	0.624	1.023	0.985–1.062	0.235
Tumor weight	1.004	1.001–1.006	0.005	1.004	1.000–1.006	0.028
**Test dataset**						
Nine genes	2.718	1.389–5.321	0.004	2.532	1.179–5.437	0.0172
Stage						
I,II	1(reference)			1(reference)		
III,IV	2.753	1.094–6.928	0.032	4.002	1.435–11.16	0.008
Age	1.015	0.981–1.050	0.396	1.021	0.984–1.06	0.264
Tumor weight	1.002	1.000–1.003	0.022	1.001	0.999–1.003	0.1001
**GEO dataset**						
Nine genes	2.718	1.774–4.165	4.384e-06	2.578	1.65–4.03	3.19e-05
Stage						
I,II	1(reference)			1(reference)		
III,IV	4.2199	2.387–7.459		4.065	2.285–7.234	1.85e-06
Age	1.006	0.988–1.025	0.492	1.017	0.999–1.037	0.0612

### Molecular Signatures of Prognostic Biomarkers

To investigate the clinical implications of the molecular signatures, we focused on the nine genes of COAD. There are six therapeutic targets of FDA-approved drugs through their associated TFs. SLC25A1 maintains mitochondrial integrity and bioenergetics in tumor cells. It prevents mitochondrial damage and circumvents mitochondrial depletion via autophagy, hence promoting proliferation (Catalina-Rodriguez et al., 2012). Several evidences implicate that SLC25A1 plays a role in cancer progression. High levels of SLC25A1 expression are associated with poor prognosis in lung cancer and estrogen receptor negative breast cancer (Georgiades et al., 1988; Jiang et al., 2017). In ovarian cancer patients, SLC25A1 mRNA levels are also associated with resistance to platinum-based chemotherapy, and blocking CTP function enhances sensitivity of cultured ovarian carcinoma cells to platinum (Georgiades et al., 1988; Jiang et al., 2017).

**FIGURE 6:**
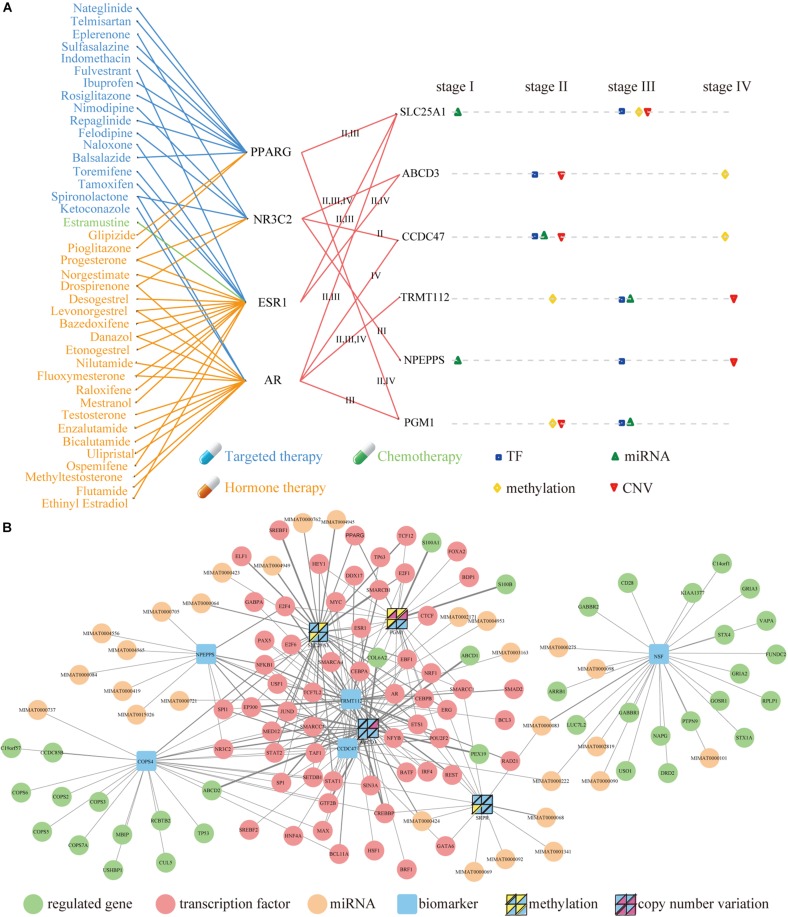
Molecular Signatures and upstream and downstream network of nine biomarkers in COAD. **(A)** The mapping between FDA-approved drugs and their related genes (left) and the influence of four factors to the nine genes (right). The blue, green and orange lines represent targeted therapy, chemotherapy and hormone therapy respectively. Square, top triangle, diamond and bottom triangle represent the regulation of TF, miRNA, methylation and CNV on related genes in four stages. **(B)** Upstream and downstream network. The thickness of the line represented the quantity of interactions in these stages. The yellow and purple triangles (inside the four panel squares) represent that the nine genes are affected by their own methylation and CNV. Each panel in the four-panel squares represents each of the four cancer stages (top left is stage I, top right is stage II, bottom left is stage III, and bottom right is stage IV).

There were 41 FDA-approved drugs related to the six therapeutic targets, and they were connected by four TFs (Figure 6A). Ethanolamine derivatives of eicosapentaenoic acid (EPA) and docosahexaenoic acid (DHA) have recently been found to induce autophagy by activating PPARG in human breast cancer cells (Rovito et al., 2015; Garay-Lugo et al., 2016). The PPARG gene is related to malignancy, which plays a vital role in the pathogenesis of multiple cancers in some clinical studies and animal models (Wang et al., 2015). AR plays a negative role in regulating the autophagy induced by celastrol, and it inhibits autophagy by transactivating mir-101 in prostate cancer cells (Guo et al., 2015). ESR1 is essential for sexual development as well as reproductive function and is involved in inducing autophagy of toxins (Chen and Xia, 2014; Tan et al., 2016).

These drugs can perform three types of treatment, including targeted therapy, hormone therapy, and chemotherapy. The Current studies have shown that the exposure to PT and balsalazide effectively inhibited the proliferation of human colon cancer HCT116 cells via inhibiting NF-κB activity and inducing apoptotic cell death. These suggest that the simultaneous administration of PT and balsalazide may provide a novel option for the treatment of colon cancer (Kim et al., 2015). The published evidence indicates that sulfasalazine prevents the development of dysplasia and CRC in patients with IBD (Eaden, 2003).

The distribution of four regulators was investigated on the six therapeutic targets in distinct stages (Figure 6A). It was found that each factor had stronger effects at different stages of cancer. In principle, their effects are present in stage II, III and IV, but there was almost no effect in stage I. A comprehensive network was constructed by integrating the upstream regulators and downstream regulated genes of the nine genes of COAD (Figure 6B and Supplementary Table S1). In final, we found that RBP play very important regulatory factors regulated autophagy-mediated CRC cell death in DEAG regulatory network (Supplementary Table S2). For example, DDX17 RNA-binding protein that regulated autophagy genes SLC25A1 and TRMT112 in the COAD is also important for the autophagy regulatory network. Similarly, POLR3A RNA-binding protein that acted as TFs performed the task of regulating autophagy genes in the READ (Supplementary Table S2). Prognosis biomarkers show the different regulation modes. It was found that more than half of the genes were regulated by TF, which existed in several stages. Then this process was systematically executed on the study of READ (Supplementary Figure S6 and Supplementary Table S1).

## Discussion

Autophagy is associated with both tumorigenic and tumor progression in CRC (Lin et al., 2014). However, the clinical significance and autophagy function in CRC remains unclear. In this study, we have revealed the expression signatures of autophagy genes regulated by multiple factors, which include TF, miRNA, promoter methylation, and CNV. Some studies have demonstrated that RNA binding proteins as TFs play a key role in the development and function of CRC (New et al., 2019). RNA binding proteins regulate the expression of thousands of transcripts and are crucial for the regulation of CRC cellular processes, such as RNA splicing, modifications, transport, and translation (Kudinov et al., 2017; Chatterji and Rustgi, 2018). For instance, Zhou B et al. found that APOBEC3G, EEF1A2, EIF5AL1, and CELF3 as RNA binding proteins may provide a good prospect for the clinical diagnosis and treatment of patients with CRC metastasis (Zhou and Guo, 2018). As other examples, PTBP1 RNA binding protein that associated with tumor metastasis in CRC tissues directly interacts with autophagy gene ATG10 and regulates ATG10 expression level (Zhou and Guo, 2018). Therefore, research on the regulation of autophagy to improve clinical outcomes is becoming increasingly important. In conclusion, some novel prognostic biomarkers associated with autophagy in CRC should be further investigated in the future.

Autophagy genes are the key components of the autophagy-mediated regulatory network. They are implicated in the occurrence and development of CRC (Hao et al., 2017). We have systematically validated the autophagy genes of differential expression, through data comparison of diverse stages for CRC. Our findings were consistent with previous reports that the signature of autophagy genes changes with different expression variation in the progression of CRC. We further analyzed the potential functional implication of autophagy genes that were specifically expressed in various periods and found that the enriched biological processes and pathways of these genes play essential roles in diverse stages of CRC. Even more, our results showed that multiple factors that regulate DEAGs are significantly different. The TF and miRNAs that regulate the autophagy genes had a very low overlap in various stages of CRC. Therefore, the modulation of autophagy genes as potential prognostic biomarkers in CRC should be further researched.

To identify potential prognostic biomarkers in CRC, we evaluated the associations between expression levels of DEAGs and the survival of the patient by employing the Cox regression analysis (Lossos et al., 2004). Multiple evidences show that SLC25A1 overexpression is associated with poor prognosis of lung cancer and estrogen receptor-negative breast cancer (Georgiades et al., 1988). These genes have a strong prognostic ability and are independent of clinical factors. As significant prognostic factors in four stages of CRC, the signature of autophagy genes will have important effects on cancer-related biological processes. However, this observation should be interpreted with caution, because there are many uncertainties in the upstream regulatory factors of autophagy. Alterations in various molecular levels could cause expression dysregulation of autophagy genes (Hao et al., 2017). Therefore, further efforts are required to elucidate the corresponding contributions of various factors in the expression signatures of the autophagy gene of CRC (Wang et al., 2016). Also, it is essential that we continue to explore the biological functions of autophagy in the context of different interactions.

In summary, we identified the potential prognostic biomarkers in CRC and described their signatures in several stages of CRC. Along with the development of cancer clinical management approaches, this study will make a significant step toward transforming them from preclinical to clinical assessments.

## Data Availability Statement

Gene and miRNA expression data, methylation data, and the clinical data of CRC patients were downloaded from TCGA (http://cancergenome.nih.gov/) (Cancer Genome Atlas Research Network, 2008). Additionally, a cohort of 177 COAD patients and 196 READ patients from GEO database (GSE17536 and GSE87211) was used as an independent external test set.

## Author Contributions

HW, KL, and JL conceived and designed the experiments, and wrote the manuscript. CZ, JJ, LW, LZ, JX, XQ, and HH collected and analyzed the data. All authors read and approved the final version of the manuscript.

## Conflict of Interest

The authors declare that the research was conducted in the absence of any commercial or financial relationships that could be construed as a potential conflict of interest.

## Footnotes

1 http://cancergenome.nih.gov/

2 www.broadinstitute.org/ccle

3 http://celldeathproteomics.uio.no./

4 http://www.hprd.org/

5 http://rna.sysu.edu.cn/chipbase/

6 http://eurbpdb.syshospital.org/

7 http://miRecords.umn.edu/miRecords

8 http://www.microrna.gr/tarbase

9 http://miRTarBase.mbc.nctu.edu.tw/
