# Supplementary material for: Identification of Autophagy-Associated Biomarkers and Corresponding Regulatory Factors in the Progression of Colorectal Cancer
Source: Front Genet. 2020 Mar 18;11:245. doi: 10.3389/fgene.2020.00245 (PMC7100633; doi:10.3389/fgene.2020.00245)
Supplement: Supplementary file 1 [file Data_Sheet_1.docx]

**Supplementary figure legends**

**Figure S1. DEAG regulatory networks in stage I-IV of COAD.** Pink nodes represent the DEAG, and blue nodes represent their downstream targeted gene.

**Figure S2. DEAG regulatory networks in stage I-IV of READ.** Pink nodes represent the DEAG, and blue nodes represent their downstream targeted gene.

**Figure S3. Analysis of DEAG regulatory network in READ.** (A) The distribution of the degree of DEAG regulatory network in stage I. (B) The distribution of the degree of DEAG regulatory network in stage II. (C) The distribution of the degree of DEAG regulatory network in stage III. (D) The distribution of the degree of DEAG regulatory network in stage IV. (E) Significantly enriched KEGG pathways of common DEAGs. (F) Significantly enriched KEGG pathways of the DEAGs in every stage.

**Figure S4. The influence of any two factors to DEAGs in the four stages of READ.** The numbers represent the proportion of the DEAGs regulated by each pair of upstream regulators in stage I (A), II (B), III (C) and IV (D). The darker color represents a larger effect.

**Figure S5. The prognostic value of the nine genes signature in READ.** (A-C) show the Kaplan-Meier survival curves for the train (A), test (B) and GEO datasets (C). The red and green lines represent the high-risk and low-risk patients respectively. (D-F) show the detailed risk score distribution of patients in the train (D), test (E) and GEO datasets (F). (G-I) show the ROC curves and AUCs of the nine gene signature predicting patients’ five-year survival in the train (G), test (H) and GEO datasets(I).

**Figure S6. Molecular Signatures and upstream and downstream network of nine biomarkers in READ.** (A) The mapping between FDA-approved drugs and their related genes (left) and the influence of four factors to the nine genes (right). The blue, green and orange lines represent targeted therapy, chemotherapy and hormone therapy respectively. Square, top triangle, diamond and bottom triangle represent the regulation of TF, miRNA, methylation and CNV on related genes in four stages. (B) Upstream and downstream network. The thickness of the line represented the quantity of interactions in these stages. The yellow and purple triangles (inside the 4 panel squares) represent that the nine genes are affected by their own methylation and CNV. Each panel in the 4-panel squares represents each of the four cancer stages (top left is stage I, top right is stage II, bottom left is stage III, and bottom right is stage IV).
