# Supplementary material for: Identification of Autophagy-Associated Biomarkers and Corresponding Regulatory Factors in the Progression of Colorectal Cancer
Source: Front Genet. 2020 Mar 18;11:245. doi: 10.3389/fgene.2020.00245 (PMC7100633; doi:10.3389/fgene.2020.00245)
Supplement: Supplementary file 2 [file Table_1.docx]

**Table S1. Upstream and downstream network of biomarkers in COAD and READ.**

| **Node1** | **Node2** | **Edge** | **Cancer Type** |
| --- | --- | --- | --- |
| ABCD3 | ABCD1 | I,II,III,IV | COAD |
| ABCD3 | ABCD2 | I,II,III,IV | COAD |
| ABCD3 | COL6A2 | I,II,III,IV | COAD |
| ABCD3 | PEX19 | I,II,III,IV | COAD |
| AR | CCDC47 | IV | COAD |
| AR | PGM1 | III | COAD |
| AR | SLC25A1 | II,III | COAD |
| AR | TRMT112 | II,III,IV | COAD |
| BATF | ABCD3 | II | COAD |
| BATF | CCDC47 | II,IV | COAD |
| BATF | TRMT112 | II | COAD |
| BCL11A | COPS4 | III | COAD |
| BCL11A | TRMT112 | III,IV | COAD |
| BCL3 | TRMT112 | III | COAD |
| BDP1 | PGM1 | II | COAD |
| BRF1 | ABCD3 | II | COAD |
| CEBPA | ABCD3 | II,IV | COAD |
| CEBPA | CCDC47 | IV | COAD |
| CEBPB | ABCD3 | II,III | COAD |
| CEBPB | CCDC47 | II | COAD |
| CEBPB | PGM1 | III | COAD |
| CEBPB | TRMT112 | III | COAD |
| COPS4 | C19orf57 | III | COAD |
| COPS4 | CCDC85B | III | COAD |
| COPS4 | COPS2 | III | COAD |
| COPS4 | COPS3 | III | COAD |
| COPS4 | COPS5 | III | COAD |
| COPS4 | COPS6 | III | COAD |
| COPS4 | COPS7A | III | COAD |
| COPS4 | CUL5 | III | COAD |
| COPS4 | MBIP | III | COAD |
| COPS4 | RCBTB2 | III | COAD |
| COPS4 | TP53 | III | COAD |
| COPS4 | USHBP1 | III | COAD |
| CREBBP | ABCD3 | II,IV | COAD |
| CREBBP | COPS4 | IV | COAD |
| CREBBP | SRPR | III | COAD |
| CREBBP | TRMT112 | I,II,III,IV | COAD |
| CTCF | SLC25A1 | III | COAD |
| CTCF | TRMT112 | II | COAD |
| DDX17 | SLC25A1 | I | COAD |
| DDX17 | TRMT112 | III,IV | COAD |
| E2F1 | PGM1 | III | COAD |
| E2F1 | SLC25A1 | II,IV | COAD |
| E2F1 | TRMT112 | III | COAD |
| E2F4 | NPEPPS | III,IV | COAD |
| E2F4 | PGM1 | III | COAD |
| E2F4 | TRMT112 | II,III,IV | COAD |
| E2F6 | ABCD3 | I,III | COAD |
| E2F6 | SLC25A1 | II,III | COAD |
| E2F6 | TRMT112 | II,III | COAD |
| EBF1 | ABCD3 | II | COAD |
| EBF1 | CCDC47 | II | COAD |
| EBF1 | PGM1 | III | COAD |
| EBF1 | SLC25A1 | II,III | COAD |
| EBF1 | TRMT112 | III | COAD |
| ELF1 | SLC25A1 | I,II,III | COAD |
| EP300 | ABCD3 | II,III | COAD |
| EP300 | CCDC47 | I,II | COAD |
| EP300 | COPS4 | IV | COAD |
| EP300 | NPEPPS | I,III | COAD |
| EP300 | SLC25A1 | III | COAD |
| EP300 | TRMT112 | I,II,III,IV | COAD |
| ERG | ABCD3 | II | COAD |
| ERG | PGM1 | III | COAD |
| ERG | SLC25A1 | II,III | COAD |
| ERG | SRPR | III | COAD |
| ERG | TRMT112 | III | COAD |
| ESR1 | ABCD3 | II,IV | COAD |
| ESR1 | SLC25A1 | II,III | COAD |
| ETS1 | ABCD3 | II | COAD |
| ETS1 | CCDC47 | II | COAD |
| ETS1 | PGM1 | III | COAD |
| ETS1 | SLC25A1 | II,III | COAD |
| ETS1 | SRPR | III | COAD |
| ETS1 | TRMT112 | I,III | COAD |
| FOXA2 | PGM1 | II | COAD |
| GABPA | TRMT112 | III | COAD |
| GATA6 | CCDC47 | IV | COAD |
| GATA6 | SRPR | III | COAD |
| GTF2B | ABCD3 | I,II,III,IV | COAD |
| GTF2B | COPS4 | IV | COAD |
| GTF2B | TRMT112 | II | COAD |
| HEY1 | PGM1 | I | COAD |
| HEY1 | SLC25A1 | II,III | COAD |
| HEY1 | TRMT112 | III | COAD |
| HNF4A | CCDC47 | IV | COAD |
| HSF1 | TRMT112 | III | COAD |
| IRF4 | ABCD3 | II | COAD |
| IRF4 | TRMT112 | III | COAD |
| JUND | ABCD3 | I,III | COAD |
| JUND | CCDC47 | IV | COAD |
| JUND | NPEPPS | II,IV | COAD |
| JUND | SLC25A1 | II,III | COAD |
| JUND | TRMT112 | IV | COAD |
| MAX | ABCD3 | II | COAD |
| MAX | CCDC47 | II | COAD |
| MAX | COPS4 | III | COAD |
| MED12 | ABCD3 | II,IV | COAD |
| MED12 | COPS4 | IV | COAD |
| MED12 | SLC25A1 | I,III | COAD |
| MED12 | TRMT112 | II,IV | COAD |
| MIMAT0000064 | SLC25A1 | II,III | COAD |
| MIMAT0000068 | SRPR | III,IV | COAD |
| MIMAT0000069 | SRPR | IV | COAD |
| MIMAT0000083 | NSF | I,III | COAD |
| MIMAT0000083 | SRPR | IV | COAD |
| MIMAT0000084 | NPEPPS | I | COAD |
| MIMAT0000090 | NSF | III | COAD |
| MIMAT0000092 | SRPR | IV | COAD |
| MIMAT0000098 | NSF | IV | COAD |
| MIMAT0000101 | NSF | II | COAD |
| MIMAT0000222 | CCDC47 | II | COAD |
| MIMAT0000222 | NSF | II | COAD |
| MIMAT0000275 | NSF | IV | COAD |
| MIMAT0000419 | NPEPPS | II | COAD |
| MIMAT0000423 | SLC25A1 | III | COAD |
| MIMAT0000424 | SRPR | III | COAD |
| MIMAT0000424 | TRMT112 | II,III | COAD |
| MIMAT0000705 | NPEPPS | I | COAD |
| MIMAT0000721 | TRMT112 | I | COAD |
| MIMAT0000737 | COPS4 | III | COAD |
| MIMAT0000762 | SLC25A1 | II,III | COAD |
| MIMAT0001341 | SRPR | IV | COAD |
| MIMAT0002171 | TRMT112 | I | COAD |
| MIMAT0002819 | NSF | III | COAD |
| MIMAT0003163 | TRMT112 | III | COAD |
| MIMAT0004556 | NPEPPS | III | COAD |
| MIMAT0004565 | NPEPPS | II | COAD |
| MIMAT0004945 | SLC25A1 | I,II,III | COAD |
| MIMAT0004949 | SLC25A1 | I,II,III | COAD |
| MIMAT0004953 | TRMT112 | IV | COAD |
| MIMAT0015026 | NPEPPS | I | COAD |
| MYC | CCDC47 | IV | COAD |
| MYC | PGM1 | II | COAD |
| MYC | SLC25A1 | IV | COAD |
| MYC | TRMT112 | III | COAD |
| NFKB1 | ABCD3 | III | COAD |
| NFKB1 | CCDC47 | II | COAD |
| NFKB1 | COPS4 | III,IV | COAD |
| NFKB1 | NPEPPS | II,III | COAD |
| NFKB1 | PGM1 | III | COAD |
| NFKB1 | SLC25A1 | III | COAD |
| NFKB1 | TRMT112 | I,II,III | COAD |
| NFYB | SLC25A1 | II,III | COAD |
| NFYB | TRMT112 | I,III,IV | COAD |
| NR3C2 | ABCD3 | II,III,IV | COAD |
| NR3C2 | CCDC47 | II | COAD |
| NR3C2 | NPEPPS | III | COAD |
| NRF1 | ABCD3 | I,II,III | COAD |
| NRF1 | CCDC47 | I | COAD |
| NSF | ARRB1 | II | COAD |
| NSF | C14orf1 | II | COAD |
| NSF | CD28 | II | COAD |
| NSF | DRD2 | II | COAD |
| NSF | FUNDC2 | II | COAD |
| NSF | GABBR1 | II | COAD |
| NSF | GABBR2 | II | COAD |
| NSF | GOSR1 | II | COAD |
| NSF | GRIA2 | II | COAD |
| NSF | GRIA3 | II | COAD |
| NSF | KIAA1377 | II | COAD |
| NSF | LUC7L2 | II | COAD |
| NSF | NAPG | II | COAD |
| NSF | PTPN9 | II | COAD |
| NSF | RPLP1 | II | COAD |
| NSF | STX1A | II | COAD |
| NSF | STX4 | II | COAD |
| NSF | USO1 | II | COAD |
| NSF | VAPA | II | COAD |
| PAX5 | ABCD3 | II | COAD |
| PAX5 | TRMT112 | III | COAD |
| PGM1 | S100A1 | I,II,III,IV | COAD |
| PGM1 | S100B | I,II,III,IV | COAD |
| POU2F2 | ABCD3 | II | COAD |
| POU2F2 | CCDC47 | II,IV | COAD |
| POU2F2 | PGM1 | III | COAD |
| POU2F2 | SRPR | III | COAD |
| POU2F2 | TRMT112 | I,III | COAD |
| PPARG | PGM1 | III,IV | COAD |
| PPARG | SLC25A1 | II,III | COAD |
| RAD21 | TRMT112 | II,III | COAD |
| REST | ABCD3 | III | COAD |
| REST | SRPR | III | COAD |
| REST | TRMT112 | II,III,IV | COAD |
| SETDB1 | COPS4 | III | COAD |
| SETDB1 | SLC25A1 | III | COAD |
| SETDB1 | TRMT112 | I | COAD |
| SIN3A | ABCD3 | II,IV | COAD |
| SIN3A | COPS4 | III | COAD |
| SIN3A | SLC25A1 | III | COAD |
| SIN3A | SRPR | IV | COAD |
| SMAD2 | TRMT112 | II,III | COAD |
| SMARCA4 | ABCD3 | I,II,III | COAD |
| SMARCA4 | CCDC47 | I | COAD |
| SMARCA4 | PGM1 | III | COAD |
| SMARCA4 | TRMT112 | II | COAD |
| SMARCB1 | SLC25A1 | I,II,III,IV | COAD |
| SMARCB1 | TRMT112 | I,II,III | COAD |
| SMARCC1 | PGM1 | II | COAD |
| SMARCC1 | SLC25A1 | I | COAD |
| SMARCC1 | SRPR | III | COAD |
| SMARCC1 | TRMT112 | IV | COAD |
| SMARCC2 | ABCD3 | II,IV | COAD |
| SMARCC2 | COPS4 | III,IV | COAD |
| SMARCC2 | PGM1 | IV | COAD |
| SMARCC2 | TRMT112 | II | COAD |
| SP1 | ABCD3 | II | COAD |
| SP1 | COPS4 | IV | COAD |
| SP1 | SLC25A1 | III | COAD |
| SP1 | TRMT112 | I,II,III | COAD |
| SPI1 | CCDC47 | II,IV | COAD |
| SPI1 | NPEPPS | I | COAD |
| SPI1 | SLC25A1 | II,III | COAD |
| SPI1 | TRMT112 | III | COAD |
| SREBF1 | SLC25A1 | I,II,III,IV | COAD |
| SREBF2 | CCDC47 | II | COAD |
| STAT1 | ABCD3 | II | COAD |
| STAT1 | COPS4 | III | COAD |
| STAT1 | PGM1 | IV | COAD |
| STAT1 | SLC25A1 | II,III | COAD |
| STAT1 | TRMT112 | III | COAD |
| STAT2 | ABCD3 | II,IV | COAD |
| STAT2 | CCDC47 | IV | COAD |
| STAT2 | COPS4 | IV | COAD |
| STAT2 | SLC25A1 | II,III | COAD |
| STAT2 | TRMT112 | III | COAD |
| TAF1 | ABCD3 | II | COAD |
| TAF1 | COPS4 | IV | COAD |
| TAF1 | SLC25A1 | I,III | COAD |
| TAF1 | TRMT112 | II,IV | COAD |
| TCF12 | PGM1 | II | COAD |
| TCF12 | SLC25A1 | I,III,IV | COAD |
| TCF7L2 | ABCD3 | II,III | COAD |
| TCF7L2 | NPEPPS | III | COAD |
| TCF7L2 | SLC25A1 | II | COAD |
| TCF7L2 | TRMT112 | II,III | COAD |
| TP63 | TRMT112 | III | COAD |
| USF1 | CCDC47 | I,II | COAD |
| USF1 | NPEPPS | II,IV | COAD |
| USF1 | TRMT112 | II,III | COAD |
| AR | RFC2 | I,II,III | READ |
| AR | RUVBL1 | II,III | READ |
| AR | SAP18 | II,III | READ |
| AR | TARS | IV | READ |
| ATF3 | RPRD1B | II | READ |
| BATF | ADNP | II,III,IV | READ |
| BATF | NAMPT | I | READ |
| BATF | RUVBL1 | II | READ |
| BCL11A | ADNP | I,II,III | READ |
| BCL11A | PMPCA | IV | READ |
| BCL3 | ACADVL | I,III | READ |
| BCL3 | ADNP | I,II,III,IV | READ |
| BCL3 | NAMPT | II,III | READ |
| BCL3 | RFC2 | III | READ |
| BDP1 | ADNP | I,II | READ |
| BDP1 | NNT | II,III | READ |
| BDP1 | RFC2 | I | READ |
| CEBPA | NAMPT | I | READ |
| CEBPA | RUVBL1 | I,III,IV | READ |
| CEBPB | ACADVL | I,II,III | READ |
| CEBPB | ADNP | I,II,III | READ |
| CEBPB | DHRS4 | I,II | READ |
| CEBPB | NAMPT | I,II | READ |
| CEBPB | PMPCA | II | READ |
| CEBPB | RUVBL1 | I | READ |
| CEBPB | SAP18 | I,III | READ |
| CEBPB | SNAPC4 | IV | READ |
| CEBPB | TARS | I | READ |
| CREBBP | ACADVL | III | READ |
| CREBBP | DHRS4 | I,II,III,IV | READ |
| CREBBP | HEATR1 | III | READ |
| CREBBP | PMPCA | I,II,III | READ |
| CREBBP | RFC2 | I,II,III,IV | READ |
| CREBBP | RPRD1B | I,II,III,IV | READ |
| CREBBP | RUVBL1 | I,II | READ |
| CREBBP | TARS | II,IV | READ |
| CTCF | ACADVL | III | READ |
| CTCF | ADNP | II,III,IV | READ |
| CTCF | HEATR1 | I | READ |
| CTCF | SNAPC4 | II | READ |
| DDX17 | ACADVL | I,II | READ |
| DDX17 | HEATR1 | II | READ |
| DDX17 | NAMPT | I | READ |
| DDX17 | PMPCA | I | READ |
| DDX17 | RFC2 | I,II,III | READ |
| DDX17 | TARS | III | READ |
| E2F1 | ACADVL | I,II,III | READ |
| E2F1 | ADNP | II,III | READ |
| E2F1 | DHRS4 | III | READ |
| E2F1 | HEATR1 | III | READ |
| E2F1 | NAMPT | I | READ |
| E2F1 | PMPCA | II,III | READ |
| E2F1 | RFC2 | I,II,III,IV | READ |
| E2F1 | RPRD1B | I,II,III | READ |
| E2F1 | RUVBL1 | I,II,III,IV | READ |
| E2F1 | SAP18 | I,II | READ |
| E2F1 | SNAPC4 | II,III | READ |
| E2F1 | TARS | II,III | READ |
| E2F4 | ACADVL | I,II,III | READ |
| E2F4 | ADNP | II,III | READ |
| E2F4 | DHRS4 | I | READ |
| E2F4 | HEATR1 | III | READ |
| E2F4 | NAMPT | I | READ |
| E2F4 | PMPCA | II,III,IV | READ |
| E2F4 | RFC2 | II,III | READ |
| E2F4 | RPRD1B | I,II,III | READ |
| E2F4 | RUVBL1 | I,II,III,IV | READ |
| E2F4 | SAP18 | I,II,III | READ |
| E2F4 | SNAPC4 | II,III,IV | READ |
| E2F4 | TARS | III | READ |
| E2F6 | ADNP | I,II,III,IV | READ |
| E2F6 | DHRS4 | I,II | READ |
| E2F6 | HEATR1 | III,IV | READ |
| E2F6 | PMPCA | III | READ |
| E2F6 | RFC2 | II,III,IV | READ |
| E2F6 | RPRD1B | I,II,III,IV | READ |
| E2F6 | RUVBL1 | III | READ |
| E2F6 | SAP18 | II,III | READ |
| E2F6 | SNAPC4 | III | READ |
| E2F6 | TARS | II | READ |
| EBF1 | ACADVL | I,II | READ |
| EBF1 | DHRS4 | II | READ |
| EBF1 | NAMPT | I | READ |
| EBF1 | NNT | III | READ |
| EBF1 | PMPCA | II,III | READ |
| EBF1 | RFC2 | II,III | READ |
| EBF1 | RUVBL1 | II,III | READ |
| EGR1 | PMPCA | II | READ |
| ELF1 | HEATR1 | II,III | READ |
| ELF1 | PMPCA | II,III | READ |
| ELF1 | RUVBL1 | III | READ |
| ELF1 | SAP18 | I,II,III,IV | READ |
| EP300 | DHRS4 | I,II | READ |
| EP300 | NAMPT | I | READ |
| EP300 | PMPCA | I,II,III,IV | READ |
| EP300 | RFC2 | I,II,III | READ |
| EP300 | RUVBL1 | I,II,III | READ |
| EP300 | SAP18 | I,II,IV | READ |
| EP300 | SNAPC4 | I,II,III | READ |
| EP300 | TARS | II,IV | READ |
| ERG | ACADVL | I,II | READ |
| ERG | DHRS4 | I,II | READ |
| ERG | HEATR1 | I | READ |
| ERG | NNT | III | READ |
| ERG | PMPCA | II,III | READ |
| ERG | RFC2 | II,III | READ |
| ERG | RPRD1B | I | READ |
| ERG | RUVBL1 | II,III | READ |
| ERG | SAP18 | II,III | READ |
| ERG | TARS | I | READ |
| ESR1 | RFC2 | II,III | READ |
| ESRRA | RFC2 | II | READ |
| ETS1 | ACADVL | II | READ |
| ETS1 | DHRS4 | II | READ |
| ETS1 | HEATR1 | II | READ |
| ETS1 | NAMPT | I | READ |
| ETS1 | PMPCA | II,III,IV | READ |
| ETS1 | RFC2 | I,II,III | READ |
| ETS1 | RUVBL1 | II,III | READ |
| ETS1 | SAP18 | I,II,III | READ |
| FOS | ACADVL | III | READ |
| FOS | SAP18 | I,II,III | READ |
| FOXP2 | ADNP | I,II,IV | READ |
| FOXP2 | NAMPT | I | READ |
| GABPA | NNT | III | READ |
| GABPA | PMPCA | II,III | READ |
| GABPA | RFC2 | II,III | READ |
| GABPA | RUVBL1 | II,III | READ |
| GABPA | SNAPC4 | II,III | READ |
| GATA2 | ACADVL | I,II,III | READ |
| GATA6 | ADNP | I | READ |
| GATA6 | NAMPT | II | READ |
| GATA6 | RPRD1B | I,III | READ |
| GATA6 | SAP18 | I | READ |
| GATA6 | SNAPC4 | II | READ |
| GTF2B | ADNP | II,III,IV | READ |
| GTF2B | HEATR1 | II,III | READ |
| GTF2B | PMPCA | II | READ |
| GTF2B | RPRD1B | II,III | READ |
| GTF2B | SAP18 | IV | READ |
| HEATR1 | XRN1 | I,II,III,IV | READ |
| HEY1 | ACADVL | II | READ |
| HEY1 | DHRS4 | II | READ |
| HEY1 | HEATR1 | IV | READ |
| HEY1 | PMPCA | II,III,IV | READ |
| HEY1 | RFC2 | II,III | READ |
| HEY1 | RUVBL1 | II | READ |
| HEY1 | SAP18 | III | READ |
| HEY1 | SNAPC4 | II | READ |
| HNF4A | ACADVL | I,II,III | READ |
| HNF4A | ADNP | I,II,III,IV | READ |
| HNF4A | DHRS4 | I,II,III,IV | READ |
| HNF4A | HEATR1 | II,III,IV | READ |
| HNF4A | RFC2 | IV | READ |
| HNF4A | RPRD1B | I,II,III,IV | READ |
| HNF4A | SAP18 | II,III | READ |
| HNF4A | TARS | I | READ |
| HSF1 | RFC2 | III | READ |
| HSF1 | SNAPC4 | III | READ |
| IRF4 | DHRS4 | II | READ |
| IRF4 | HEATR1 | II | READ |
| IRF4 | PMPCA | II,III | READ |
| IRF4 | RFC2 | II,III | READ |
| IRF4 | RUVBL1 | II,III | READ |
| JUN | ADNP | I,II | READ |
| JUN | NNT | IV | READ |
| JUN | PMPCA | II | READ |
| JUN | RFC2 | II,III | READ |
| JUN | RPRD1B | II | READ |
| JUND | ACADVL | III | READ |
| JUND | ADNP | III,IV | READ |
| JUND | HEATR1 | II,III,IV | READ |
| JUND | RPRD1B | I,III,IV | READ |
| MAX | ADNP | I,II,IV | READ |
| MAX | DHRS4 | II,III,IV | READ |
| MAX | HEATR1 | II,III,IV | READ |
| MAX | NNT | II | READ |
| MAX | PMPCA | III | READ |
| MAX | RPRD1B | II,IV | READ |
| MAX | RUVBL1 | III | READ |
| MAX | SAP18 | II,III | READ |
| MED12 | ADNP | I,II,III,IV | READ |
| MED12 | DHRS4 | I,II,III,IV | READ |
| MED12 | PMPCA | I,II | READ |
| MED12 | RFC2 | I,II,III,IV | READ |
| MED12 | SNAPC4 | II | READ |
| MIMAT0000076 | ADNP | II | READ |
| MIMAT0000080 | PMPCA | III | READ |
| MIMAT0000092 | HEATR1 | I | READ |
| MIMAT0000096 | RFC2 | II | READ |
| MIMAT0000096 | SORD | I,III | READ |
| MIMAT0000104 | NNT | IV | READ |
| MIMAT0000271 | GABARAP | I | READ |
| MIMAT0000273 | SORD | I | READ |
| MIMAT0000461 | GABARAP | III | READ |
| MIMAT0000731 | SORD | I,III | READ |
| MIMAT0000772 | SORD | I | READ |
| MIMAT0002173 | SORD | I | READ |
| MIMAT0002808 | NNT | III | READ |
| MIMAT0003260 | NNT | III | READ |
| MIMAT0003882 | RFC2 | II | READ |
| MIMAT0004504 | ACADVL | IV | READ |
| MIMAT0004609 | PMPCA | III | READ |
| MIMAT0004613 | NNT | III | READ |
| MIMAT0004949 | SAP18 | III | READ |
| MIMAT0004950 | TARS | I | READ |
| MIMAT0004959 | SORD | II | READ |
| MIMAT0005459 | NNT | II | READ |
| MIMAT0005572 | NNT | IV | READ |
| MIMAT0005893 | NNT | III | READ |
| MIMAT0015378 | TARS | I | READ |
| MIMAT0016915 | PMPCA | III | READ |
| MIMAT0018077 | NNT | IV | READ |
| MYC | ACADVL | I,II,III | READ |
| MYC | ADNP | I,II,III | READ |
| MYC | DHRS4 | III | READ |
| MYC | HEATR1 | II,III,IV | READ |
| MYC | RFC2 | II,III | READ |
| MYC | RPRD1B | I,II,III | READ |
| MYC | RUVBL1 | I,II,III,IV | READ |
| MYC | SAP18 | III | READ |
| MYC | TARS | III | READ |
| NANOG | ADNP | II,IV | READ |
| NANOG | HEATR1 | II | READ |
| NANOG | NAMPT | II | READ |
| NANOG | PMPCA | II | READ |
| NANOG | SNAPC4 | II,III | READ |
| NFKB1 | ADNP | II | READ |
| NFKB1 | NNT | III | READ |
| NFKB1 | PMPCA | III | READ |
| NFKB1 | RFC2 | II,III | READ |
| NFKB1 | SAP18 | I,II,III | READ |
| NFKB1 | SNAPC4 | III | READ |
| NFYB | RUVBL1 | III | READ |
| NR3C2 | ACADVL | II,III | READ |
| NR3C2 | ADNP | II,III | READ |
| NR3C2 | PMPCA | III | READ |
| NRF1 | TARS | III | READ |
| PAX5 | NNT | IV | READ |
| PAX5 | PMPCA | II | READ |
| PAX5 | RFC2 | III | READ |
| PAX5 | RUVBL1 | III | READ |
| PAX5 | SAP18 | II,III,IV | READ |
| PAX5 | SNAPC4 | I | READ |
| PAX5 | TARS | I,III | READ |
| PBX3 | HEATR1 | II | READ |
| PBX3 | PMPCA | II,III | READ |
| PBX3 | RFC2 | II | READ |
| PBX3 | RUVBL1 | II | READ |
| PBX3 | SNAPC4 | II | READ |
| POLR3A | HEATR1 | I,II,III | READ |
| POLR3A | RFC2 | I | READ |
| POLR3A | RPRD1B | I,II,III,IV | READ |
| POU2F2 | DHRS4 | II | READ |
| POU2F2 | PMPCA | II,III | READ |
| POU2F2 | RFC2 | I,II,III | READ |
| POU2F2 | RUVBL1 | II,III | READ |
| POU2F2 | SAP18 | I,II,III,IV | READ |
| POU2F2 | TARS | III | READ |
| POU5F1 | ADNP | II | READ |
| PPARG | ACADVL | I,III | READ |
| RAD21 | ADNP | III | READ |
| RAD21 | HEATR1 | III | READ |
| RAD21 | RFC2 | III | READ |
| RAD21 | SNAPC4 | II | READ |
| RAD21 | TARS | III | READ |
| REST | ADNP | II,IV | READ |
| REST | DHRS4 | II,III,IV | READ |
| REST | HEATR1 | I,II | READ |
| REST | NNT | I,III | READ |
| REST | PMPCA | I,II,III,IV | READ |
| REST | RFC2 | I,II,III,IV | READ |
| REST | RPRD1B | II,IV | READ |
| REST | RUVBL1 | II,III,IV | READ |
| REST | SAP18 | I,II | READ |
| RUNX2 | TARS | IV | READ |
| RUVBL1 | ACTL6A | I,II,III,IV | READ |
| RUVBL1 | DKC1 | I,II,III,IV | READ |
| RUVBL1 | RUVBL2 | I,II,III,IV | READ |
| SETDB1 | ADNP | II,III,IV | READ |
| SETDB1 | DHRS4 | III,IV | READ |
| SETDB1 | HEATR1 | I,II,III,IV | READ |
| SETDB1 | NAMPT | II | READ |
| SETDB1 | RFC2 | I,II | READ |
| SETDB1 | SNAPC4 | II,III | READ |
| SIN3A | ACADVL | III | READ |
| SIN3A | NAMPT | I | READ |
| SIN3A | PMPCA | II,III,IV | READ |
| SIN3A | RFC2 | II | READ |
| SIN3A | SAP18 | I,II,IV | READ |
| SIN3A | SNAPC4 | II | READ |
| SIX5 | ACADVL | II | READ |
| SIX5 | ADNP | II | READ |
| SIX5 | RFC2 | III | READ |
| SIX5 | RPRD1B | I,II,III | READ |
| SIX5 | SNAPC4 | I,II | READ |
| SMAD2 | ACADVL | I,II | READ |
| SMAD2 | ADNP | I,II,III | READ |
| SMAD2 | DHRS4 | I | READ |
| SMAD2 | SNAPC4 | IV | READ |
| SMAD4 | ADNP | I,II | READ |
| SMARCA4 | ADNP | II | READ |
| SMARCA4 | HEATR1 | II,III | READ |
| SMARCA4 | NAMPT | II | READ |
| SMARCA4 | SAP18 | I | READ |
| SMARCA4 | SNAPC4 | II,III | READ |
| SMARCB1 | ACADVL | II | READ |
| SMARCB1 | ADNP | I,III | READ |
| SMARCB1 | DHRS4 | I,II,III | READ |
| SMARCB1 | PMPCA | II | READ |
| SMARCB1 | RFC2 | I,II,III | READ |
| SMARCB1 | RPRD1B | I,II | READ |
| SMARCB1 | RUVBL1 | III | READ |
| SMARCB1 | TARS | II | READ |
| SMARCC1 | ACADVL | I,II,III | READ |
| SMARCC1 | ADNP | I,II,III,IV | READ |
| SMARCC1 | HEATR1 | I,II,III | READ |
| SMARCC1 | NNT | II | READ |
| SMARCC1 | PMPCA | I,II | READ |
| SMARCC1 | RFC2 | I | READ |
| SMARCC1 | RPRD1B | I,II,III,IV | READ |
| SMARCC1 | RUVBL1 | II,III | READ |
| SMARCC1 | SNAPC4 | II | READ |
| SMARCC1 | TARS | I,III | READ |
| SMARCC2 | HEATR1 | II,III | READ |
| SMARCC2 | NAMPT | I,II | READ |
| SMARCC2 | PMPCA | I,II | READ |
| SMARCC2 | RFC2 | I,II,IV | READ |
| SMARCC2 | RPRD1B | II | READ |
| SMARCC2 | RUVBL1 | I | READ |
| SMARCC2 | SAP18 | I | READ |
| SNAPC4 | POU2F1 | II,III,IV | READ |
| SP1 | NAMPT | I | READ |
| SP1 | PMPCA | I,II,III | READ |
| SP1 | RFC2 | I,II,III,IV | READ |
| SP1 | RUVBL1 | I,II,III | READ |
| SP1 | SAP18 | I,II,IV | READ |
| SP1 | SNAPC4 | II | READ |
| SPDEF | HEATR1 | I,II,III | READ |
| SPDEF | RUVBL1 | I | READ |
| SPDEF | TARS | I | READ |
| SPI1 | ADNP | II | READ |
| SPI1 | HEATR1 | II | READ |
| SPI1 | PMPCA | II,III | READ |
| SPI1 | RFC2 | III | READ |
| SPI1 | RUVBL1 | II,III | READ |
| SREBF1 | ACADVL | I,II,III | READ |
| SREBF1 | DHRS4 | II,III | READ |
| SREBF1 | NAMPT | II | READ |
| SREBF1 | SAP18 | I,II | READ |
| SREBF2 | NAMPT | I | READ |
| STAT1 | ADNP | II,IV | READ |
| STAT1 | HEATR1 | I,II | READ |
| STAT1 | RPRD1B | IV | READ |
| STAT1 | RUVBL1 | II,III | READ |
| STAT1 | SAP18 | II,III | READ |
| STAT2 | ADNP | II,III | READ |
| STAT2 | HEATR1 | II | READ |
| STAT2 | PMPCA | II,III | READ |
| STAT2 | RFC2 | I,II,III | READ |
| STAT2 | SAP18 | I,II,III,IV | READ |
| STAT2 | TARS | II,III | READ |
| TAF1 | ACADVL | III | READ |
| TAF1 | ADNP | I,II,III | READ |
| TAF1 | DHRS4 | II,III | READ |
| TAF1 | HEATR1 | I,II,III | READ |
| TAF1 | NAMPT | II | READ |
| TAF1 | NNT | II | READ |
| TAF1 | PMPCA | I,II | READ |
| TAF1 | RFC2 | I,II | READ |
| TAF1 | RPRD1B | I,II,III | READ |
| TAF1 | SNAPC4 | II,III | READ |
| TCF12 | PMPCA | II,III | READ |
| TCF12 | RFC2 | II,IV | READ |
| TCF12 | SNAPC4 | III | READ |
| TCF7L2 | ACADVL | I,III | READ |
| TCF7L2 | ADNP | I,II,III | READ |
| TCF7L2 | DHRS4 | II | READ |
| TCF7L2 | HEATR1 | II,III | READ |
| TCF7L2 | RFC2 | III | READ |
| TCF7L2 | RPRD1B | I,II,III,IV | READ |
| TCF7L2 | RUVBL1 | III | READ |
| TCF7L2 | SAP18 | I | READ |
| TCF7L2 | TARS | I | READ |
| TFAP2A | ACADVL | II,III | READ |
| TFAP2A | PMPCA | II | READ |
| TFAP2A | SNAPC4 | I | READ |
| TFAP2A | TARS | I,IV | READ |
| TFAP2C | ACADVL | IV | READ |
| TFAP2C | PMPCA | III | READ |
| TFAP2C | RUVBL1 | I | READ |
| TFAP2C | TARS | II | READ |
| TP63 | ADNP | II,IV | READ |
| USF1 | ACADVL | I,II,III | READ |
| USF1 | ADNP | I,II | READ |
| USF1 | DHRS4 | II | READ |
| USF1 | PMPCA | I,II | READ |
| USF1 | RPRD1B | II,IV | READ |
| USF1 | RUVBL1 | III | READ |
| USF1 | TARS | III | READ |
| ZBTB33 | ADNP | I,II,III | READ |
| ZBTB33 | HEATR1 | I,II,III | READ |
| ZBTB33 | TARS | I | READ |
