# Supplementary material for: Identification of Autophagy-Associated Biomarkers and Corresponding Regulatory Factors in the Progression of Colorectal Cancer
Source: Front Genet. 2020 Mar 18;11:245. doi: 10.3389/fgene.2020.00245 (PMC7100633; doi:10.3389/fgene.2020.00245)
Supplement: Supplementary file 3 [file Table_2.docx]

Supplementary Table S2. RNA binding proteins and the autophagy genes they regulate in COAD and READ.

| **RPB** | **Node** | **Cancer_type** |
| --- | --- | --- |
| COPS4 | C19orf57 | COAD |
| COPS4 | CCDC85B | COAD |
| COPS4 | COPS2 | COAD |
| COPS4 | COPS3 | COAD |
| COPS4 | COPS5 | COAD |
| COPS4 | COPS6 | COAD |
| COPS4 | COPS7A | COAD |
| COPS4 | CUL5 | COAD |
| COPS4 | MBIP | COAD |
| COPS4 | RCBTB2 | COAD |
| COPS4 | TP53 | COAD |
| COPS4 | USHBP1 | COAD |
| CREBBP | ABCD3 | COAD |
| CREBBP | COPS4 | COAD |
| CREBBP | SRPR | COAD |
| CREBBP | TRMT112 | COAD |
| CTCF | SLC25A1 | COAD |
| CTCF | TRMT112 | COAD |
| DDX17 | SLC25A1 | COAD |
| DDX17 | TRMT112 | COAD |
| GTF2B | ABCD3 | COAD |
| GTF2B | COPS4 | COAD |
| GTF2B | TRMT112 | COAD |
| MED12 | ABCD3 | COAD |
| MED12 | COPS4 | COAD |
| MED12 | SLC25A1 | COAD |
| MED12 | TRMT112 | COAD |
| NSF | ARRB1 | COAD |
| NSF | C14orf1 | COAD |
| NSF | CD28 | COAD |
| NSF | DRD2 | COAD |
| NSF | FUNDC2 | COAD |
| NSF | GABBR1 | COAD |
| NSF | GABBR2 | COAD |
| NSF | GOSR1 | COAD |
| NSF | GRIA2 | COAD |
| NSF | GRIA3 | COAD |
| NSF | KIAA1377 | COAD |
| NSF | LUC7L2 | COAD |
| NSF | NAPG | COAD |
| NSF | PTPN9 | COAD |
| NSF | RPLP1 | COAD |
| NSF | STX1A | COAD |
| NSF | STX4 | COAD |
| NSF | USO1 | COAD |
| NSF | VAPA | COAD |
| SETDB1 | COPS4 | COAD |
| SETDB1 | SLC25A1 | COAD |
| SETDB1 | TRMT112 | COAD |
| SIN3A | ABCD3 | COAD |
| SIN3A | COPS4 | COAD |
| SIN3A | SLC25A1 | COAD |
| SIN3A | SRPR | COAD |
| SMARCA4 | ABCD3 | COAD |
| SMARCA4 | CCDC47 | COAD |
| SMARCA4 | PGM1 | COAD |
| SMARCA4 | TRMT112 | COAD |
| SMARCC1 | PGM1 | COAD |
| SMARCC1 | SLC25A1 | COAD |
| SMARCC1 | SRPR | COAD |
| SMARCC1 | TRMT112 | COAD |
| SMARCC2 | ABCD3 | COAD |
| SMARCC2 | COPS4 | COAD |
| SMARCC2 | PGM1 | COAD |
| SMARCC2 | TRMT112 | COAD |
| STAT1 | ABCD3 | COAD |
| STAT1 | COPS4 | COAD |
| STAT1 | PGM1 | COAD |
| STAT1 | SLC25A1 | COAD |
| STAT1 | TRMT112 | COAD |
| CCDC47 | AR | COAD |
| CCDC47 | BATF | COAD |
| CCDC47 | CEBPA | COAD |
| CCDC47 | CEBPB | COAD |
| CCDC47 | EBF1 | COAD |
| CCDC47 | EP300 | COAD |
| CCDC47 | ETS1 | COAD |
| CCDC47 | GATA6 | COAD |
| CCDC47 | HNF4A | COAD |
| CCDC47 | JUND | COAD |
| CCDC47 | MAX | COAD |
| CCDC47 | MIMAT0000222 | COAD |
| CCDC47 | MYC | COAD |
| CCDC47 | NFKB1 | COAD |
| CCDC47 | NR3C2 | COAD |
| CCDC47 | NRF1 | COAD |
| CCDC47 | POU2F2 | COAD |
| CCDC47 | SMARCA4 | COAD |
| CCDC47 | SPI1 | COAD |
| CCDC47 | SREBF2 | COAD |
| CCDC47 | STAT2 | COAD |
| CCDC47 | USF1 | COAD |
| TRMT112 | AR | COAD |
| TRMT112 | BATF | COAD |
| TRMT112 | BCL11A | COAD |
| TRMT112 | BCL3 | COAD |
| TRMT112 | CEBPB | COAD |
| TRMT112 | CREBBP | COAD |
| TRMT112 | CTCF | COAD |
| TRMT112 | DDX17 | COAD |
| TRMT112 | E2F1 | COAD |
| TRMT112 | E2F4 | COAD |
| TRMT112 | E2F6 | COAD |
| TRMT112 | EBF1 | COAD |
| TRMT112 | EP300 | COAD |
| TRMT112 | ERG | COAD |
| TRMT112 | ETS1 | COAD |
| TRMT112 | GABPA | COAD |
| TRMT112 | GTF2B | COAD |
| TRMT112 | HEY1 | COAD |
| TRMT112 | HSF1 | COAD |
| TRMT112 | IRF4 | COAD |
| TRMT112 | JUND | COAD |
| TRMT112 | MED12 | COAD |
| TRMT112 | MIMAT0000424 | COAD |
| TRMT112 | MIMAT0000721 | COAD |
| TRMT112 | MIMAT0002171 | COAD |
| TRMT112 | MIMAT0003163 | COAD |
| TRMT112 | MIMAT0004953 | COAD |
| TRMT112 | MYC | COAD |
| TRMT112 | NFKB1 | COAD |
| TRMT112 | NFYB | COAD |
| TRMT112 | PAX5 | COAD |
| TRMT112 | POU2F2 | COAD |
| TRMT112 | RAD21 | COAD |
| TRMT112 | REST | COAD |
| TRMT112 | SETDB1 | COAD |
| TRMT112 | SMAD2 | COAD |
| TRMT112 | SMARCA4 | COAD |
| TRMT112 | SMARCB1 | COAD |
| TRMT112 | SMARCC1 | COAD |
| TRMT112 | SMARCC2 | COAD |
| TRMT112 | SP1 | COAD |
| TRMT112 | SPI1 | COAD |
| TRMT112 | STAT1 | COAD |
| TRMT112 | STAT2 | COAD |
| TRMT112 | TAF1 | COAD |
| TRMT112 | TCF7L2 | COAD |
| TRMT112 | TP63 | COAD |
| TRMT112 | USF1 | COAD |
| COPS4 | BCL11A | COAD |
| COPS4 | CREBBP | COAD |
| COPS4 | EP300 | COAD |
| COPS4 | GTF2B | COAD |
| COPS4 | MAX | COAD |
| COPS4 | MED12 | COAD |
| COPS4 | MIMAT0000737 | COAD |
| COPS4 | NFKB1 | COAD |
| COPS4 | SETDB1 | COAD |
| COPS4 | SIN3A | COAD |
| COPS4 | SMARCC2 | COAD |
| COPS4 | SP1 | COAD |
| COPS4 | STAT1 | COAD |
| COPS4 | STAT2 | COAD |
| COPS4 | TAF1 | COAD |
| COPS2 | COPS4 | COAD |
| COPS3 | COPS4 | COAD |
| NPEPPS | E2F4 | COAD |
| NPEPPS | EP300 | COAD |
| NPEPPS | JUND | COAD |
| NPEPPS | MIMAT0000084 | COAD |
| NPEPPS | MIMAT0000419 | COAD |
| NPEPPS | MIMAT0000705 | COAD |
| NPEPPS | MIMAT0004556 | COAD |
| NPEPPS | MIMAT0004565 | COAD |
| NPEPPS | MIMAT0015026 | COAD |
| NPEPPS | NFKB1 | COAD |
| NPEPPS | NR3C2 | COAD |
| NPEPPS | SPI1 | COAD |
| NPEPPS | TCF7L2 | COAD |
| NPEPPS | USF1 | COAD |
| NSF | MIMAT0000083 | COAD |
| NSF | MIMAT0000090 | COAD |
| NSF | MIMAT0000098 | COAD |
| NSF | MIMAT0000101 | COAD |
| NSF | MIMAT0000222 | COAD |
| NSF | MIMAT0000275 | COAD |
| NSF | MIMAT0002819 | COAD |
| LUC7L2 | NSF | COAD |
| RPLP1 | NSF | COAD |
| USO1 | NSF | COAD |
| VAPA | NSF | COAD |
| CREBBP | ACADVL | READ |
| CREBBP | DHRS4 | READ |
| CREBBP | HEATR1 | READ |
| CREBBP | PMPCA | READ |
| CREBBP | RFC2 | READ |
| CREBBP | RPRD1B | READ |
| CREBBP | RUVBL1 | READ |
| CREBBP | TARS | READ |
| CTCF | ACADVL | READ |
| CTCF | ADNP | READ |
| CTCF | HEATR1 | READ |
| CTCF | SNAPC4 | READ |
| DDX17 | ACADVL | READ |
| DDX17 | HEATR1 | READ |
| DDX17 | NAMPT | READ |
| DDX17 | PMPCA | READ |
| DDX17 | RFC2 | READ |
| DDX17 | TARS | READ |
| GTF2B | ADNP | READ |
| GTF2B | HEATR1 | READ |
| GTF2B | PMPCA | READ |
| GTF2B | RPRD1B | READ |
| GTF2B | SAP18 | READ |
| HEATR1 | XRN1 | READ |
| MED12 | ADNP | READ |
| MED12 | DHRS4 | READ |
| MED12 | PMPCA | READ |
| MED12 | RFC2 | READ |
| MED12 | SNAPC4 | READ |
| POLR3A | HEATR1 | READ |
| POLR3A | RFC2 | READ |
| POLR3A | RPRD1B | READ |
| RUVBL1 | ACTL6A | READ |
| RUVBL1 | DKC1 | READ |
| RUVBL1 | RUVBL2 | READ |
| SETDB1 | ADNP | READ |
| SETDB1 | DHRS4 | READ |
| SETDB1 | HEATR1 | READ |
| SETDB1 | NAMPT | READ |
| SETDB1 | RFC2 | READ |
| SETDB1 | SNAPC4 | READ |
| SIN3A | ACADVL | READ |
| SIN3A | NAMPT | READ |
| SIN3A | PMPCA | READ |
| SIN3A | RFC2 | READ |
| SIN3A | SAP18 | READ |
| SIN3A | SNAPC4 | READ |
| SMARCA4 | ADNP | READ |
| SMARCA4 | HEATR1 | READ |
| SMARCA4 | NAMPT | READ |
| SMARCA4 | SAP18 | READ |
| SMARCA4 | SNAPC4 | READ |
| SMARCC1 | ACADVL | READ |
| SMARCC1 | ADNP | READ |
| SMARCC1 | HEATR1 | READ |
| SMARCC1 | NNT | READ |
| SMARCC1 | PMPCA | READ |
| SMARCC1 | RFC2 | READ |
| SMARCC1 | RPRD1B | READ |
| SMARCC1 | RUVBL1 | READ |
| SMARCC1 | SNAPC4 | READ |
| SMARCC1 | TARS | READ |
| SMARCC2 | HEATR1 | READ |
| SMARCC2 | NAMPT | READ |
| SMARCC2 | PMPCA | READ |
| SMARCC2 | RFC2 | READ |
| SMARCC2 | RPRD1B | READ |
| SMARCC2 | RUVBL1 | READ |
| SMARCC2 | SAP18 | READ |
| STAT1 | ADNP | READ |
| STAT1 | HEATR1 | READ |
| STAT1 | RPRD1B | READ |
| STAT1 | RUVBL1 | READ |
| STAT1 | SAP18 | READ |
| RFC2 | AR | READ |
| RFC2 | BCL3 | READ |
| RFC2 | BDP1 | READ |
| RFC2 | CREBBP | READ |
| RFC2 | DDX17 | READ |
| RFC2 | E2F1 | READ |
| RFC2 | E2F4 | READ |
| RFC2 | E2F6 | READ |
| RFC2 | EBF1 | READ |
| RFC2 | EP300 | READ |
| RFC2 | ERG | READ |
| RFC2 | ESR1 | READ |
| RFC2 | ESRRA | READ |
| RFC2 | ETS1 | READ |
| RFC2 | GABPA | READ |
| RFC2 | HEY1 | READ |
| RFC2 | HNF4A | READ |
| RFC2 | HSF1 | READ |
| RFC2 | IRF4 | READ |
| RFC2 | JUN | READ |
| RFC2 | MED12 | READ |
| RFC2 | MIMAT0000096 | READ |
| RFC2 | MIMAT0003882 | READ |
| RFC2 | MYC | READ |
| RFC2 | NFKB1 | READ |
| RFC2 | PAX5 | READ |
| RFC2 | PBX3 | READ |
| RFC2 | POLR3A | READ |
| RFC2 | POU2F2 | READ |
| RFC2 | RAD21 | READ |
| RFC2 | REST | READ |
| RFC2 | SETDB1 | READ |
| RFC2 | SIN3A | READ |
| RFC2 | SIX5 | READ |
| RFC2 | SMARCB1 | READ |
| RFC2 | SMARCC1 | READ |
| RFC2 | SMARCC2 | READ |
| RFC2 | SP1 | READ |
| RFC2 | SPI1 | READ |
| RFC2 | STAT2 | READ |
| RFC2 | TAF1 | READ |
| RFC2 | TCF12 | READ |
| RFC2 | TCF7L2 | READ |
| RUVBL1 | AR | READ |
| RUVBL1 | BATF | READ |
| RUVBL1 | CEBPA | READ |
| RUVBL1 | CEBPB | READ |
| RUVBL1 | CREBBP | READ |
| RUVBL1 | E2F1 | READ |
| RUVBL1 | E2F4 | READ |
| RUVBL1 | E2F6 | READ |
| RUVBL1 | EBF1 | READ |
| RUVBL1 | ELF1 | READ |
| RUVBL1 | EP300 | READ |
| RUVBL1 | ERG | READ |
| RUVBL1 | ETS1 | READ |
| RUVBL1 | GABPA | READ |
| RUVBL1 | HEY1 | READ |
| RUVBL1 | IRF4 | READ |
| RUVBL1 | MAX | READ |
| RUVBL1 | MYC | READ |
| RUVBL1 | NFYB | READ |
| RUVBL1 | PAX5 | READ |
| RUVBL1 | PBX3 | READ |
| RUVBL1 | POU2F2 | READ |
| RUVBL1 | REST | READ |
| RUVBL1 | SMARCB1 | READ |
| RUVBL1 | SMARCC1 | READ |
| RUVBL1 | SMARCC2 | READ |
| RUVBL1 | SP1 | READ |
| RUVBL1 | SPDEF | READ |
| RUVBL1 | SPI1 | READ |
| RUVBL1 | STAT1 | READ |
| RUVBL1 | TCF7L2 | READ |
| RUVBL1 | TFAP2C | READ |
| RUVBL1 | USF1 | READ |
| SAP18 | AR | READ |
| SAP18 | CEBPB | READ |
| SAP18 | E2F1 | READ |
| SAP18 | E2F4 | READ |
| SAP18 | E2F6 | READ |
| SAP18 | ELF1 | READ |
| SAP18 | EP300 | READ |
| SAP18 | ERG | READ |
| SAP18 | ETS1 | READ |
| SAP18 | FOS | READ |
| SAP18 | GATA6 | READ |
| SAP18 | GTF2B | READ |
| SAP18 | HEY1 | READ |
| SAP18 | HNF4A | READ |
| SAP18 | MAX | READ |
| SAP18 | MIMAT0004949 | READ |
| SAP18 | MYC | READ |
| SAP18 | NFKB1 | READ |
| SAP18 | PAX5 | READ |
| SAP18 | POU2F2 | READ |
| SAP18 | REST | READ |
| SAP18 | SIN3A | READ |
| SAP18 | SMARCA4 | READ |
| SAP18 | SMARCC2 | READ |
| SAP18 | SP1 | READ |
| SAP18 | SREBF1 | READ |
| SAP18 | STAT1 | READ |
| SAP18 | STAT2 | READ |
| SAP18 | TCF7L2 | READ |
| TARS | AR | READ |
| TARS | CEBPB | READ |
| TARS | CREBBP | READ |
| TARS | DDX17 | READ |
| TARS | E2F1 | READ |
| TARS | E2F4 | READ |
| TARS | E2F6 | READ |
| TARS | EP300 | READ |
| TARS | ERG | READ |
| TARS | HNF4A | READ |
| TARS | MIMAT0004950 | READ |
| TARS | MIMAT0015378 | READ |
| TARS | MYC | READ |
| TARS | NRF1 | READ |
| TARS | PAX5 | READ |
| TARS | POU2F2 | READ |
| TARS | RAD21 | READ |
| TARS | RUNX2 | READ |
| TARS | SMARCB1 | READ |
| TARS | SMARCC1 | READ |
| TARS | SPDEF | READ |
| TARS | STAT2 | READ |
| TARS | TCF7L2 | READ |
| TARS | TFAP2A | READ |
| TARS | TFAP2C | READ |
| TARS | USF1 | READ |
| TARS | ZBTB33 | READ |
| ADNP | BATF | READ |
| ADNP | BCL11A | READ |
| ADNP | BCL3 | READ |
| ADNP | BDP1 | READ |
| ADNP | CEBPB | READ |
| ADNP | CTCF | READ |
| ADNP | E2F1 | READ |
| ADNP | E2F4 | READ |
| ADNP | E2F6 | READ |
| ADNP | FOXP2 | READ |
| ADNP | GATA6 | READ |
| ADNP | GTF2B | READ |
| ADNP | HNF4A | READ |
| ADNP | JUN | READ |
| ADNP | JUND | READ |
| ADNP | MAX | READ |
| ADNP | MED12 | READ |
| ADNP | MIMAT0000076 | READ |
| ADNP | MYC | READ |
| ADNP | NANOG | READ |
| ADNP | NFKB1 | READ |
| ADNP | NR3C2 | READ |
| ADNP | POU5F1 | READ |
| ADNP | RAD21 | READ |
| ADNP | REST | READ |
| ADNP | SETDB1 | READ |
| ADNP | SIX5 | READ |
| ADNP | SMAD2 | READ |
| ADNP | SMAD4 | READ |
| ADNP | SMARCA4 | READ |
| ADNP | SMARCB1 | READ |
| ADNP | SMARCC1 | READ |
| ADNP | SPI1 | READ |
| ADNP | STAT1 | READ |
| ADNP | STAT2 | READ |
| ADNP | TAF1 | READ |
| ADNP | TCF7L2 | READ |
| ADNP | TP63 | READ |
| ADNP | USF1 | READ |
| ADNP | ZBTB33 | READ |
| PMPCA | BCL11A | READ |
| PMPCA | CEBPB | READ |
| PMPCA | CREBBP | READ |
| PMPCA | DDX17 | READ |
| PMPCA | E2F1 | READ |
| PMPCA | E2F4 | READ |
| PMPCA | E2F6 | READ |
| PMPCA | EBF1 | READ |
| PMPCA | EGR1 | READ |
| PMPCA | ELF1 | READ |
| PMPCA | EP300 | READ |
| PMPCA | ERG | READ |
| PMPCA | ETS1 | READ |
| PMPCA | GABPA | READ |
| PMPCA | GTF2B | READ |
| PMPCA | HEY1 | READ |
| PMPCA | IRF4 | READ |
| PMPCA | JUN | READ |
| PMPCA | MAX | READ |
| PMPCA | MED12 | READ |
| PMPCA | MIMAT0000080 | READ |
| PMPCA | MIMAT0004609 | READ |
| PMPCA | MIMAT0016915 | READ |
| PMPCA | NANOG | READ |
| PMPCA | NFKB1 | READ |
| PMPCA | NR3C2 | READ |
| PMPCA | PAX5 | READ |
| PMPCA | PBX3 | READ |
| PMPCA | POU2F2 | READ |
| PMPCA | REST | READ |
| PMPCA | SIN3A | READ |
| PMPCA | SMARCB1 | READ |
| PMPCA | SMARCC1 | READ |
| PMPCA | SMARCC2 | READ |
| PMPCA | SP1 | READ |
| PMPCA | SPI1 | READ |
| PMPCA | STAT2 | READ |
| PMPCA | TAF1 | READ |
| PMPCA | TCF12 | READ |
| PMPCA | TFAP2A | READ |
| PMPCA | TFAP2C | READ |
| PMPCA | USF1 | READ |
| ACADVL | BCL3 | READ |
| ACADVL | CEBPB | READ |
| ACADVL | CREBBP | READ |
| ACADVL | CTCF | READ |
| ACADVL | DDX17 | READ |
| ACADVL | E2F1 | READ |
| ACADVL | E2F4 | READ |
| ACADVL | EBF1 | READ |
| ACADVL | ERG | READ |
| ACADVL | ETS1 | READ |
| ACADVL | FOS | READ |
| ACADVL | GATA2 | READ |
| ACADVL | HEY1 | READ |
| ACADVL | HNF4A | READ |
| ACADVL | JUND | READ |
| ACADVL | MIMAT0004504 | READ |
| ACADVL | MYC | READ |
| ACADVL | NR3C2 | READ |
| ACADVL | PPARG | READ |
| ACADVL | SIN3A | READ |
| ACADVL | SIX5 | READ |
| ACADVL | SMAD2 | READ |
| ACADVL | SMARCB1 | READ |
| ACADVL | SMARCC1 | READ |
| ACADVL | SREBF1 | READ |
| ACADVL | TAF1 | READ |
| ACADVL | TCF7L2 | READ |
| ACADVL | TFAP2A | READ |
| ACADVL | TFAP2C | READ |
| ACADVL | USF1 | READ |
| NNT | BDP1 | READ |
| NNT | EBF1 | READ |
| NNT | ERG | READ |
| NNT | GABPA | READ |
| NNT | JUN | READ |
| NNT | MAX | READ |
| NNT | MIMAT0000104 | READ |
| NNT | MIMAT0002808 | READ |
| NNT | MIMAT0003260 | READ |
| NNT | MIMAT0004613 | READ |
| NNT | MIMAT0005459 | READ |
| NNT | MIMAT0005572 | READ |
| NNT | MIMAT0005893 | READ |
| NNT | MIMAT0018077 | READ |
| NNT | NFKB1 | READ |
| NNT | PAX5 | READ |
| NNT | REST | READ |
| NNT | SMARCC1 | READ |
| NNT | TAF1 | READ |
| HEATR1 | CREBBP | READ |
| HEATR1 | CTCF | READ |
| HEATR1 | DDX17 | READ |
| HEATR1 | E2F1 | READ |
| HEATR1 | E2F4 | READ |
| HEATR1 | E2F6 | READ |
| HEATR1 | ELF1 | READ |
| HEATR1 | ERG | READ |
| HEATR1 | ETS1 | READ |
| HEATR1 | GTF2B | READ |
| HEATR1 | HEY1 | READ |
| HEATR1 | HNF4A | READ |
| HEATR1 | IRF4 | READ |
| HEATR1 | JUND | READ |
| HEATR1 | MAX | READ |
| HEATR1 | MIMAT0000092 | READ |
| HEATR1 | MYC | READ |
| HEATR1 | NANOG | READ |
| HEATR1 | PBX3 | READ |
| HEATR1 | POLR3A | READ |
| HEATR1 | RAD21 | READ |
| HEATR1 | REST | READ |
| HEATR1 | SETDB1 | READ |
| HEATR1 | SMARCA4 | READ |
| HEATR1 | SMARCC1 | READ |
| HEATR1 | SMARCC2 | READ |
| HEATR1 | SPDEF | READ |
| HEATR1 | SPI1 | READ |
| HEATR1 | STAT1 | READ |
| HEATR1 | STAT2 | READ |
| HEATR1 | TAF1 | READ |
| HEATR1 | TCF7L2 | READ |
| HEATR1 | ZBTB33 | READ |
| XRN1 | HEATR1 | READ |
| DKC1 | RUVBL1 | READ |
| RUVBL2 | RUVBL1 | READ |

Supplementary Table S2.2. The percentage of each factor in the COAD

|  | Stage.I | Stage.II | Stage.III | Stage.IV |
| --- | --- | --- | --- | --- |
| TF | 0.426518 | 0.642857 | 0.601108 | 0.376238 |
| miRNA | 0.086262 | 0.027094 | 0.026316 | 0.089109 |
| methylation | 0.009585 | 0.004926 | 0.00831 | 0.015842 |
| CNV | 0.015974 | 0.003695 | 0.00554 | 0.035644 |
| two factors | 0.183706 | 0.220443 | 0.243767 | 0.152475 |
| three factors | 0.014377 | 0.013547 | 0.019391 | 0.017822 |
| four factors | 0.001597 | 0 | 0 | 0 |
| others | 0.261981 | 0.087438 | 0.095568 | 0.312871 |

Supplementary Table S2.3. The percentage of each factor in the READ

|  | Stage.I | Stage.II | Stage.III | Stage.IV |
| --- | --- | --- | --- | --- |
| TF | 0.234513 | 0.222772 | 0.167382 | 0.199005 |
| miRNA | 0.053097 | 0.019802 | 0.030043 | 0.059701 |
| methylation | 0.022124 | 0.039604 | 0.008584 | 0.039801 |
| CNV | 0.137168 | 0.108911 | 0.06867 | 0.119403 |
| two factors | 0.265487 | 0.356436 | 0.506438 | 0.308458 |
| three factors | 0.070796 | 0.123762 | 0.120172 | 0.049751 |
| four factors | 0 | 0.024752 | 0.021459 | 0 |
| others | 0.216814 | 0.10396 | 0.077253 | 0.223881 |
