# Supplementary material for: Identification of Autophagy-Associated Biomarkers and Corresponding Regulatory Factors in the Progression of Colorectal Cancer
Source: Front Genet. 2020 Mar 18;11:245. doi: 10.3389/fgene.2020.00245 (PMC7100633; doi:10.3389/fgene.2020.00245)
Supplement: Supplementary file 4 [file Table_3.docx]

Supplementary Table S3. The percentage of each factor in the COAD

|  | Stage.I | Stage.II | Stage.III | Stage.IV |
| --- | --- | --- | --- | --- |
| TF | 0.426518 | 0.642857 | 0.601108 | 0.376238 |
| miRNA | 0.086262 | 0.027094 | 0.026316 | 0.089109 |
| methylation | 0.009585 | 0.004926 | 0.00831 | 0.015842 |
| CNV | 0.015974 | 0.003695 | 0.00554 | 0.035644 |
| two factors | 0.183706 | 0.220443 | 0.243767 | 0.152475 |
| three factors | 0.014377 | 0.013547 | 0.019391 | 0.017822 |
| four factors | 0.001597 | 0 | 0 | 0 |
| others | 0.261981 | 0.087438 | 0.095568 | 0.312871 |

The percentage of each factor in the READ

|  | Stage.I | Stage.II | Stage.III | Stage.IV |
| --- | --- | --- | --- | --- |
| TF | 0.234513 | 0.222772 | 0.167382 | 0.199005 |
| miRNA | 0.053097 | 0.019802 | 0.030043 | 0.059701 |
| methylation | 0.022124 | 0.039604 | 0.008584 | 0.039801 |
| CNV | 0.137168 | 0.108911 | 0.06867 | 0.119403 |
| two factors | 0.265487 | 0.356436 | 0.506438 | 0.308458 |
| three factors | 0.070796 | 0.123762 | 0.120172 | 0.049751 |
| four factors | 0 | 0.024752 | 0.021459 | 0 |
| others | 0.216814 | 0.10396 | 0.077253 | 0.223881 |
