# Supplementary figures and images for: Identification of Autophagy-Associated Biomarkers and Corresponding Regulatory Factors in the Progression of Colorectal Cancer
Source: Front Genet. 2020 Mar 18;11:245. doi: 10.3389/fgene.2020.00245 (PMC7100633; doi:10.3389/fgene.2020.00245)

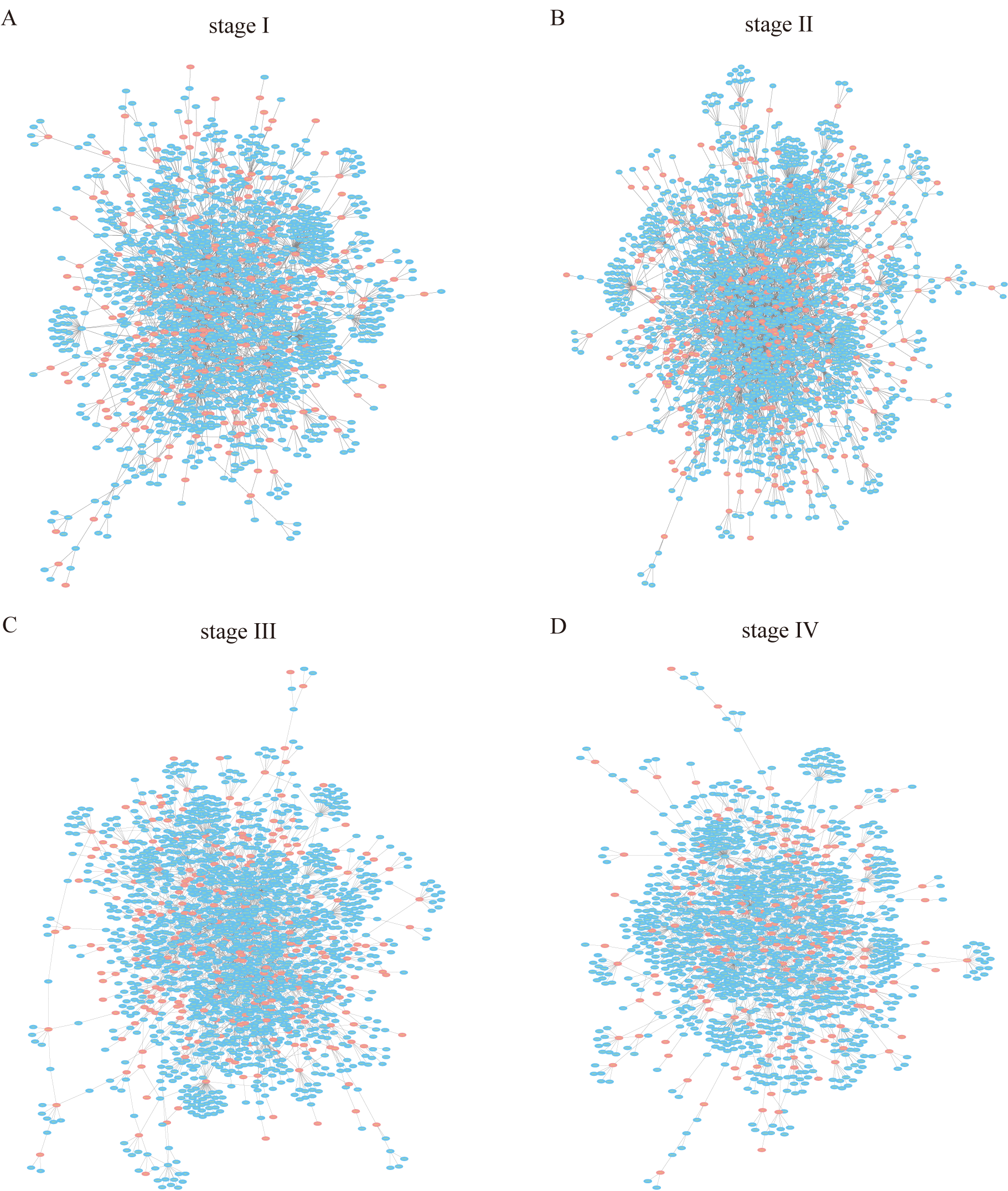

Supplement: Supplementary file 5 [file Image_1.TIF]

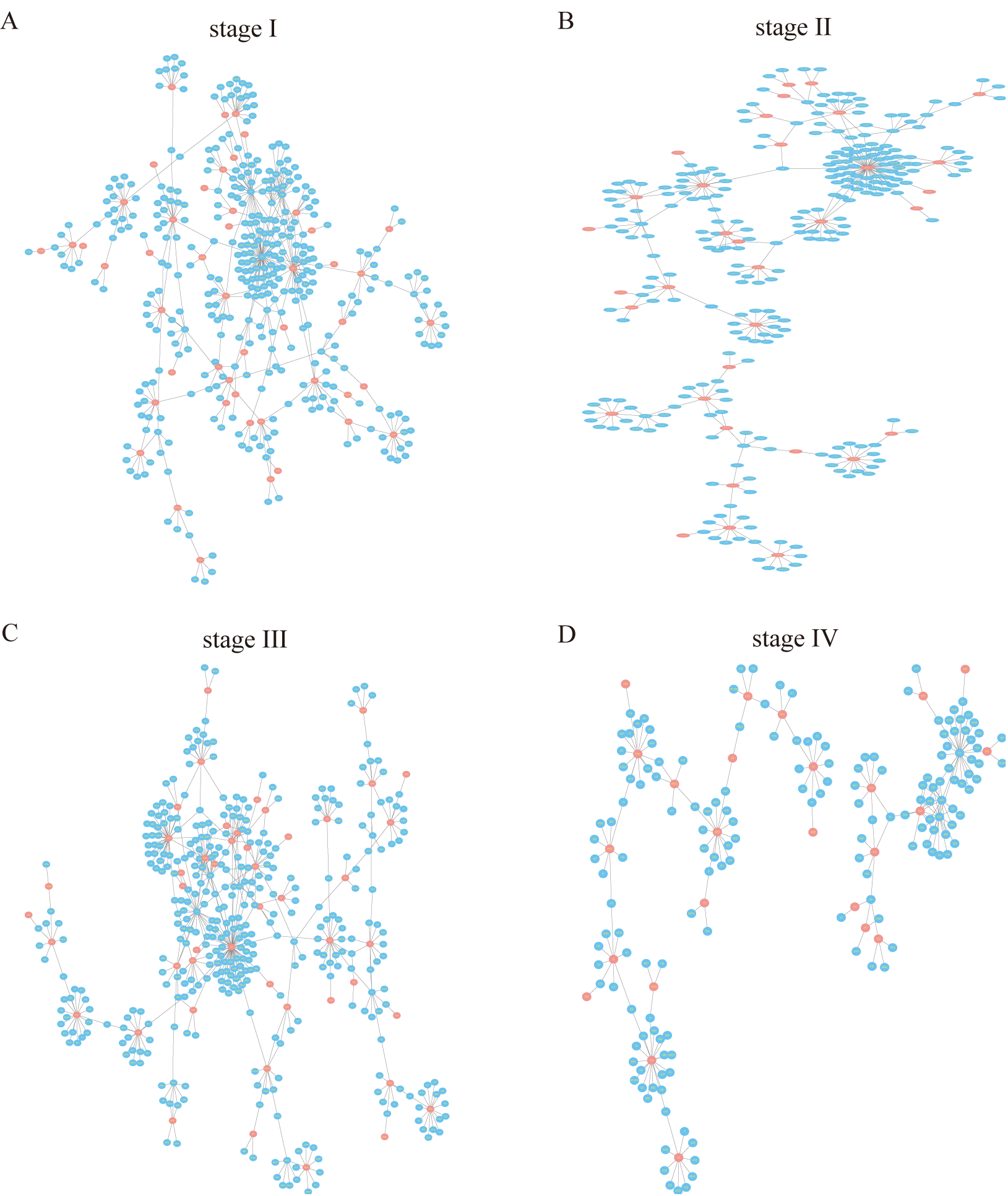

Supplement: Supplementary file 6 [file Image_2.TIF]

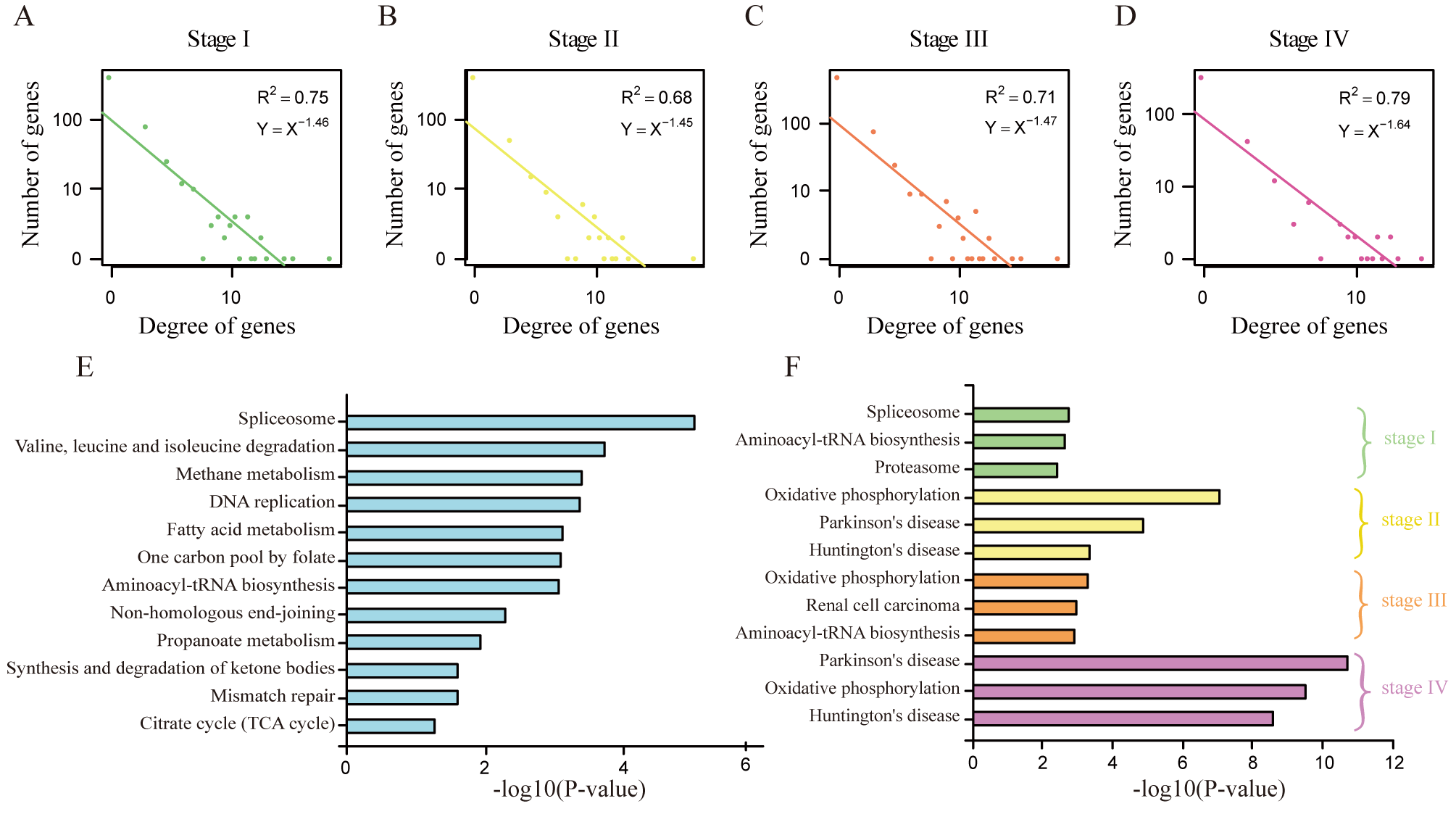

Supplement: Supplementary file 7 [file Image_3.TIF]

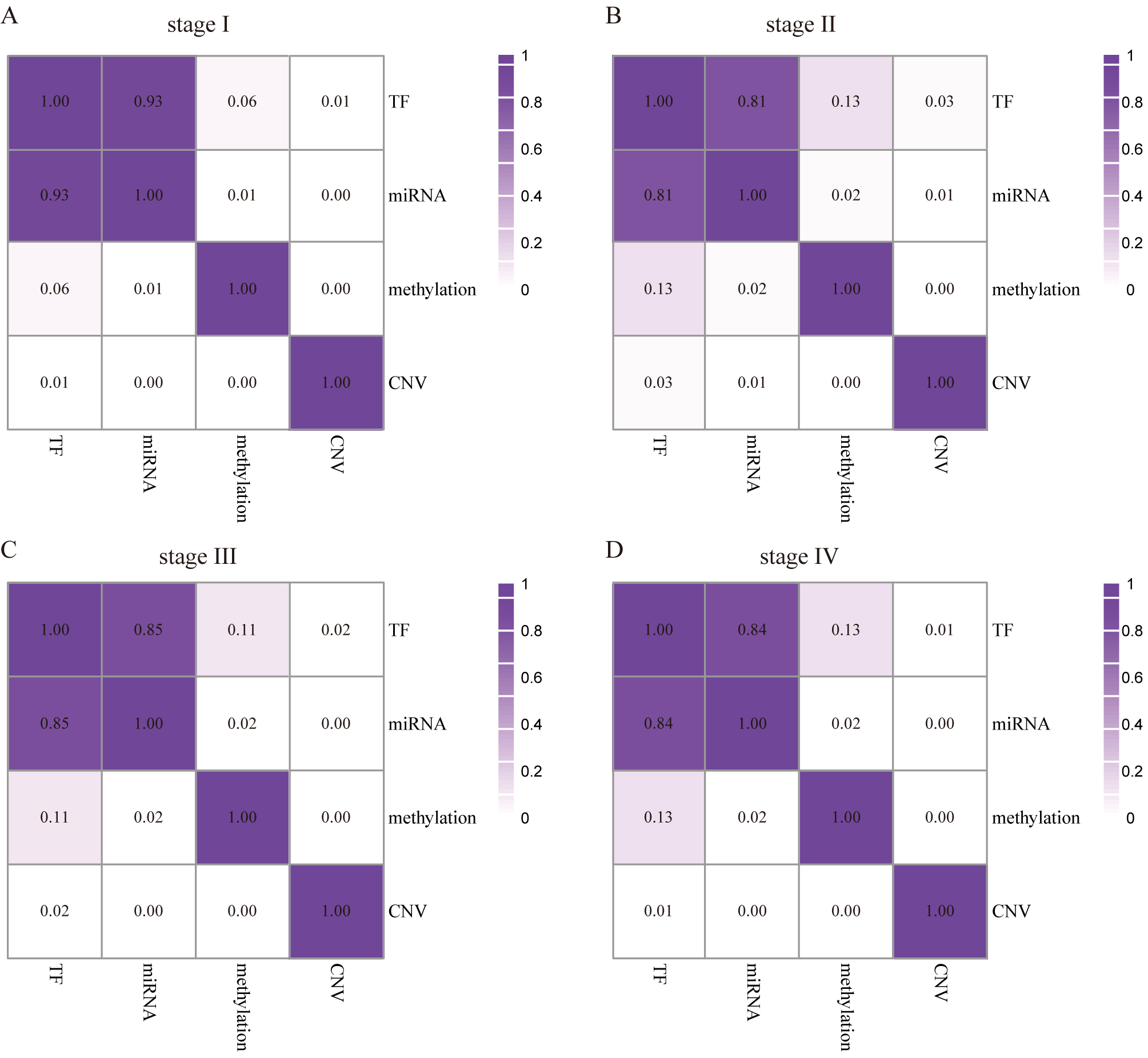

Supplement: Supplementary file 8 [file Image_4.TIF]

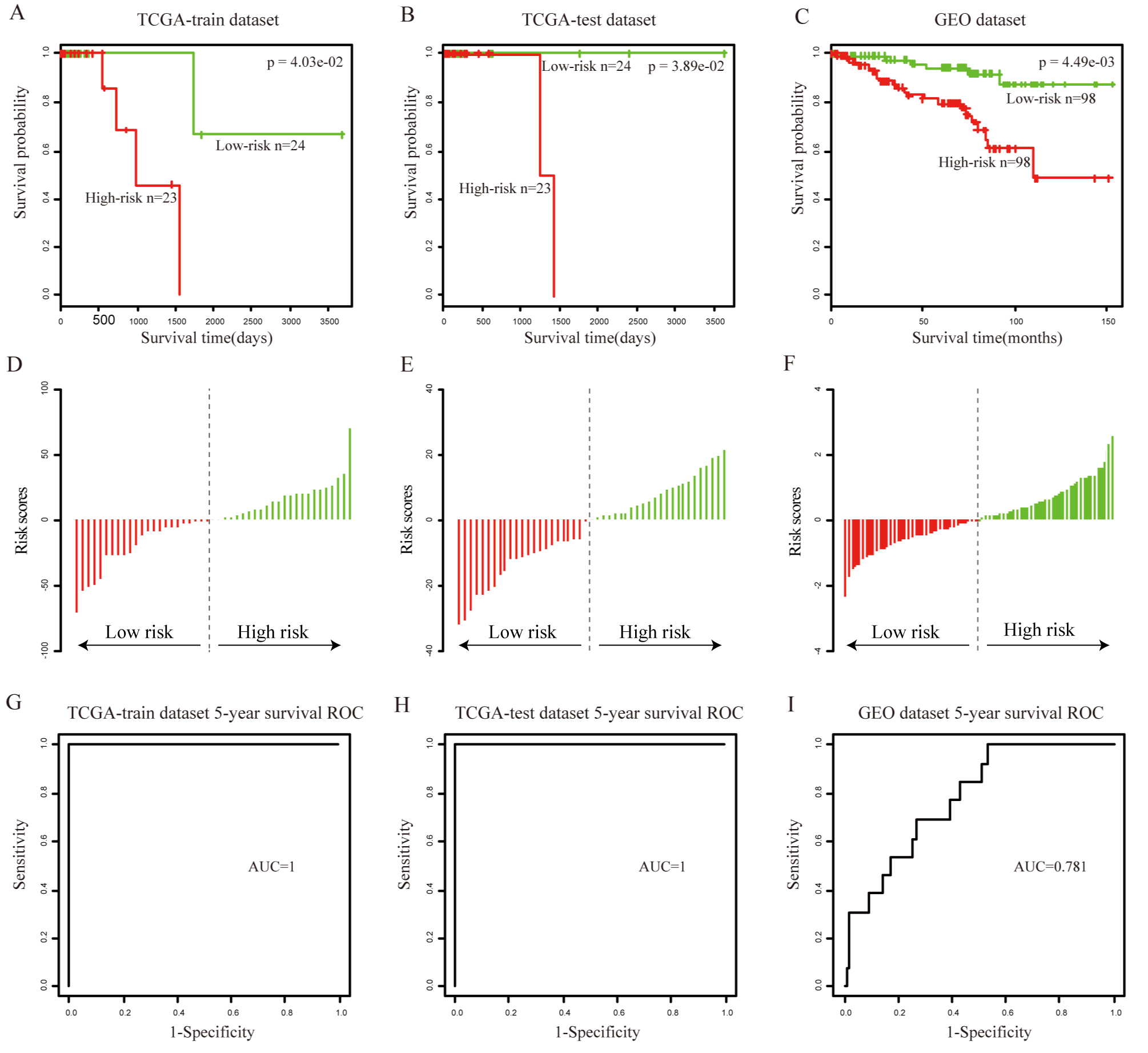

Supplement: Supplementary file 9 [file Image_5.TIF]

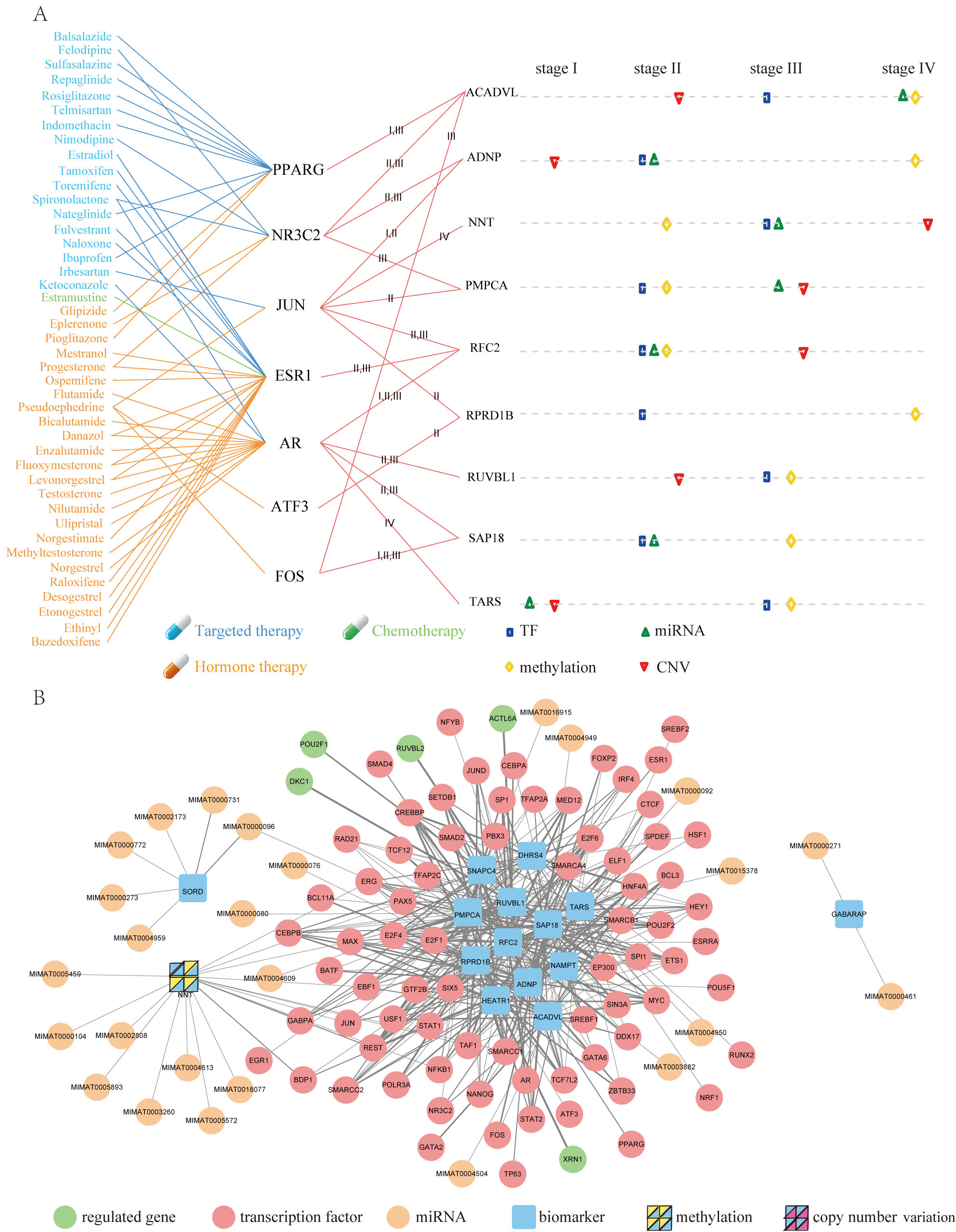

Supplement: Supplementary file 10 [file Image_6.TIF]
